# Global Burden and Gender Disparities in Head and Neck Cancers Among Adults Aged 40–64, 1990–2021: A Systematic Analysis From the Global Burden of Disease Study 2021

**DOI:** 10.1002/cnr2.70287

**Published:** 2025-08-20

**Authors:** Zhuoding Luo, Yihan Huang, Renjing Ye, Min Yin

**Affiliations:** ^1^ Department of Otorhinolaryngology The First Affiliated Hospital of Nanjing Medical University Nanjing China; ^2^ The First School of Clinical Medicine, Nanjing Medical University Nanjing China

**Keywords:** age‐standardized rate, gender disparities, Global Burden of Disease, head and neck cancer, risk factors

## Abstract

**Background:**

Head and neck cancer (HNC) is a significant global health concern, with incidence rising after the age of 40. This study aims to analyze the trends in prevalence, incidence, mortality, and disability‐adjusted life years (DALYs), focusing on regional and gender differences.

**Methods:**

This research utilized data from the Global Burden of Disease (GBD) 2021 study. Key metrics such as the age‐standardized prevalence rate (ASPR), age‐standardized incidence rate (ASIR), age‐standardized mortality rate (ASMR), and age‐standardized disability‐adjusted life years rate (ASDR) were analyzed. These metrics were used to examine trends from 1990 to 2021, focusing on gender and regional differences, and projections were made using the Nordpred method to predict future disease burdens up to 2045. The analysis covered 204 countries and regions and focused on cancers of the lip and oral cavity, nasopharynx, other pharynx, and larynx.

**Results:**

From 1990 to 2021, the prevalence of HNC in the 40–64 age group nearly doubled, yet the ASPR remained stable. In contrast, ASIR, ASMR, and ASDR showed a decreasing trend. The analysis revealed significant gender differences, with males generally exhibiting higher ASIR, ASMR, and ASDR than females. However, the prevalence and incidence rates among females showed a faster increase in certain regions, particularly in lower SDI countries.

**Conclusion:**

The study concludes that while the overall burden of HNC has shifted with a stable ASPR and decreasing ASIR, ASMR, and ASDR, gender‐specific and region‐specific strategies are essential to effectively address the risk factors associated with HNC.

**Abbreviations**
AAPC: average annual percentage change
APC: annual percentage change
ASDR: age‐standardized disability‐adjusted life years rate
ASIR: age‐standardized incidence rate
ASMR: age‐standardized mortality rate
ASPR: age‐standardized prevalence rate
CI: confidence interval
DALYs: disability‐adjusted life years
EBV: Epstein–Barr virus
GBD: Global Burden of Disease
HNC: head and neck cancer
HNSCC: head and neck squamous cell carcinoma
HPV: human papillomavirus
NPC: nasopharyngeal cancer
PAF: population attributable fraction
SDI: sociodemographic index

## 1 Background

Head and neck cancer (HNC) is a common malignant tumor primarily occurring in the epithelial lining of the oral cavity, pharynx, and larynx [1]. They have similar anatomical and pathological characteristics. The majority of HNC cases are squamous cell carcinomas (HNSCC), with a significantly increased incidence after the age of 40 [2]. These cancers are commonly associated with risk factors such as tobacco use, alcohol consumption, and human papillomavirus (HPV) infection [3, 4]. In 2022, there were approximately 120 416 new cases of nasopharyngeal cancer, 188 960 cases of laryngeal cancer, and 389 485 cases of lip and oral cavity cancer worldwide, accounting for 0.6%, 0.9%, and 2% of all cancer cases, respectively [5]. The incidence and mortality rates of these cancers vary significantly across different regions and populations, closely linked to various environmental, genetic, and lifestyle factors [6]. HNC was once among the top 10 most common cancers in men, but its ranking among all cancers has gradually declined in recent years [5]. This trend is attributed to the rapidly increasing incidence of cancers, particularly prostate, thyroid, and colorectal cancers.

The global incidence of HNC has generally been on the rise over the past few decades, especially in certain developed countries, with specific subtypes of the disease driving this trend [7]. High‐incidence areas are primarily found in Southeast Asia, South Asia, and parts of Africa [8]. Factors such as socioeconomic status, dietary practices, and access to healthcare play a significant role in influencing these rates [9, 10]. Interestingly, in some developed nations, changes in lifestyle and the introduction of HPV vaccination have begun to shift these trends [6]. While the incidence of traditional tobacco‐related HNC is declining, cases of HPV‐related HNC are increasing [11, 12]. For instance, in the United States, the overall incidence of HNC slightly decreased by 0.22% annually between 2002 and 2012, but HPV‐related oropharyngeal cancers rose by 2.5% each year [13]. In contrast, regions like Japan and Taiwan are seeing a continued rise in HNC cases. In Taiwan, HPV‐related HNC has seen an annual growth rate of 6.9%, particularly among males aged 40–50 [14].

Overall, the incidence of HNC is significantly higher in males than in females around the world. However, in recent years, certain regions have observed a faster increase in HNC cases among females than males [15]. This trend is particularly evident in parts of Europe and Asia, likely due to changes in smoking and alcohol consumption habits among females in these areas [16].

The global incidence of HNC exhibits significant regional, gender, and cancer subtype variations. HPV infection is increasingly becoming a major driver of the rising incidence of certain types of HNC, particularly oropharyngeal cancer [7]. Although overall HNC rates have declined in some regions, the upward trend in HPV‐related cancers remains concerning [17]. However, there has yet to be a comprehensive study that specifically describes the global burden of HNC and its long‐term trends, particularly in terms of differences between genders and across countries with varying levels of socioeconomic development.

This study used data from the Global Burden of Disease (GBD) database to examine the burden of HNC among middle‐aged adults (ages 40–64) worldwide from 1990 to 2021. We focused on key metrics such as prevalence, incidence, mortality, and disability‐adjusted life years (DALYs), with particular emphasis on regional and gender differences in incidence and mortality rates. Additionally, we projected trends in disease burden from 2022 to 2045 and evaluated the factors that could potentially influence HNC incidence moving forward.

## 2 Methods

### 2.1 Data Sources

The GBD 2021 project estimated the prevalence, incidence, mortality, and DALYs associated with 371 diseases and injuries, and 88 risk factors in 204 countries and territories from 1990 to 2021. The input sources followed the Guidelines for Accurate and Transparent Health Estimates Reporting (GATHER) statement. Data on the prevalence, incidence, mortality, and DALYs of HNC across different ages, genders, and regions, as well as the attributable risk factors, were collected through the Global Health Data Exchange (https://vizhub.healthdata.org/gbd‐results/). The results are presented with a 95% uncertainty interval (UI). We extracted the annual incidence, mortality, and DALYs data of lip and oral cavity cancer, nasopharynx cancer, other pharynx cancer, and larynx cancer from 1990 to 2021.

DALYs are a core measure of the total burden of disease, combining the loss of longevity due to premature death and the loss of healthy quality of life due to disease. Taking into account cultural and social differences, we defined middle‐aged HNC patients as those aged 40–64 years, divided into five age groups: 40–44, 45–49, 50–54, 55–59, and 60–64 years.

We used the International Classification of Diseases and Injuries‐10 diagnostic codes to distinguish lip and oral cavity cancer (C00–C07, C08–C08.9), nasopharynx cancer (C11–C11.9), other pharynx cancer (C09–C10.9, C12–C13.9), and larynx cancer (C32–C32.9).

The sociodemographic index (SDI) ranges from 0 (the lowest) to 1 (the highest) and divides 204 countries and territories into five levels: low, low‐middle, middle, high‐middle, and high, which is calculated by per capita income, average educational attainment, and total fertility rate. Higher values indicate a higher level of socioeconomic development of the country or region [18].

### 2.2 Statistical Analysis

To ensure data comparability across different times, countries, and regions, we used age‐standardized rates (ASRs) as objective indicators to quantify trends in HNC. ASRs are summary measures that adjust age‐specific rates to a standard population structure, allowing for fair comparisons of disease burden across populations or time periods by eliminating the effects of varying age distributions. These indicators include the age‐standardized prevalence rate (ASPR), age‐standardized incidence rate (ASIR), age‐standardized mortality rate (ASMR), and age‐standardized DALYs rate (ASDR). The Joinpoint regression model is a statistical method for analyzing trends in disease incidence and mortality by fitting changes in the trends over multiple periods of time. This study used Joinpoint software (version 5.0.2; National Cancer Institute, Bethesda, MD, USA) for trend analysis, where the joinpoint regression model applies the *Z*‐test to hypothesize the presence of joinpoints. Initially, the model assumes no joinpoints (H0: 0 joinpoints) and tests against the alternative hypothesis that at least one joinpoint exists (H1: at least 1 joinpoint). If H0 is rejected, further tests are conducted to determine if there are statistically significant differences between one joinpoint and multiple joinpoints, and so on. The annual percentage change (APC) and average annual percentage change (AAPC) were calculated, with a significance level set at *p* < 0.05. If the AAPC value and the lower limit of its 95% confidence interval (CI) are both greater than 0, the ASRs are considered to be significantly increasing. Conversely, if both are less than 0, the ASRs are considered to be decreasing. If the 95% CI includes 0, the ASRs are considered stable [19].

The formula for calculating ASR is as follows: $\mathrm{ASR}=\frac{\sum_{i=1}^{n}\left(R_{i}\times W_{i}\right)}{\sum_{i=1}^{n}W_{i}},$ where *R*_*i*_ is the crude incidence or mortality rate in the *i*‐th age group, *W*_*i*_ is the standard population proportion corresponding to the *i*‐th age group (usually based on the age distribution of a standard population, such as the world standard population or the population distribution of a specific region), and *n* is the number of age groups. AAPC is calculated as a weighted average of the annual percentage variations from the joinpoint regression analysis. DALYs were calculated as the sum of years of life lost due to premature mortality (YLLs) and years lived with disability (YLDs): DALYs=YLLs+YLDs. YLLs were derived from the number of deaths multiplied by the standard life expectancy at the age of death. YLDs were estimated by multiplying the prevalence of each condition by a corresponding disability weight reflecting its severity.

The Nordpred method projects future disease incidence and mortality rates by adjusting for population aging and demographic changes using age–period–cohort (APC) modeling. It first models historical data to estimate trends, then projects these trends into the future, considering potential shifts in risk factors and demographics. This method is valued for its ability to produce reliable long‐term predictions by incorporating demographic changes and associated uncertainties.

Calculations and graph drawings were performed using R (version 4.3.2, Posit PBC, Boston, MA, USA).

## 3 Results

### 3.1 Global Trends

**FIGURE 1:**
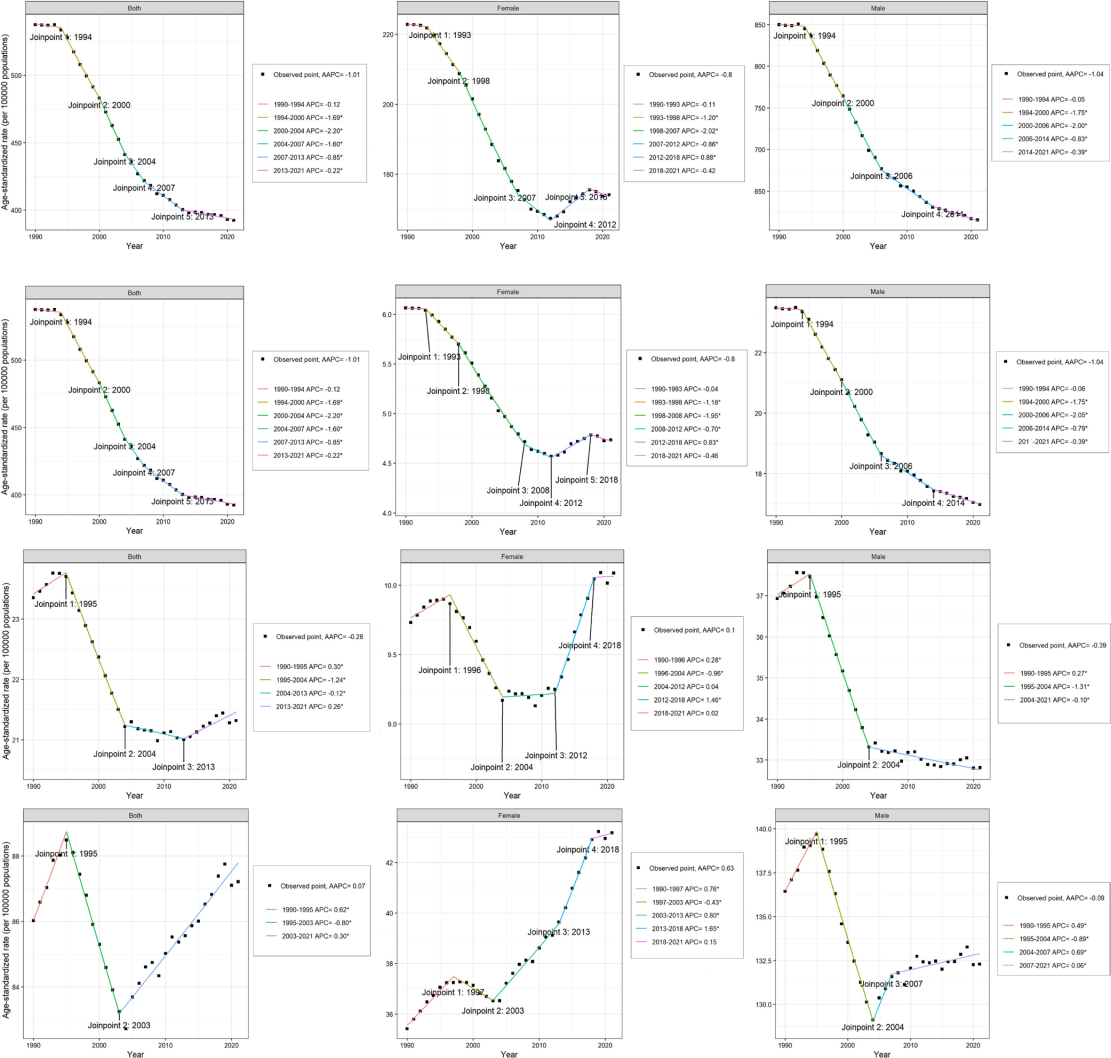
Joinpoint regression analysis showing temporal trends in age‐standardized DALYs rate (ASDR), mortality rate (ASMR), incidence rate (ASIR), and prevalence rate (ASPR) of head and neck cancer (HNC) from 1990 to 2021, stratified by gender. The four panels display trends from top to bottom in the following order: ASDR, ASMR, ASIR, and ASPR. The figure illustrates gender differences and turning points in HNC burden over time.

From 1990 to 2021, the prevalence of HNC among individuals aged 40–64 increased from 0.918 million to 1.887 million. However, the ASR in this age group remained relatively stable, at 86.0 (95% UI: 81.2–91.3, Table S1) per 100 000 in 1990 compared to 87.2 (79.7–95.5) per 100 000 in 2021, with an AAPC of 0.07% (95% CI: 0.01–0.12). During this period, the prevalence of specific HNC types showed different trends: lip and oral cavity cancer increased from 31.2 to 37.0 per 100 000, nasopharynx cancer from 13.3 to 14.4 per 100 000, and other pharynx cancers from 6.11 to 9.71 per 100 000, while larynx cancer decreased from 35.5 to 26.1 per 100 000 (Figure 1 bottom). The proportion of HNC patients aged 40–64 relative to the total number of HNC cases decreased from 58.8% to 54.0%.

In contrast to prevalence, the incidence, mortality, and DALYs of HNC in the 40–64 age group showed a downward trend. The ASIR per 100 000 decreased from 23.4 (21.9–25.0) to 21.3 (19.4–23.3) per 100 000, with an AAPC of −0.28% (−0.33% to −0.23%) (Figure 1 lower). The ASMR per 100 000 declined from 14.8 (13.6–16.0) to 10.8 (9.73–11.8) per 100 000, with an AAPC of −1.01% (−1.09% to −0.92%) (Figure 1 upper). The ASDR decreased from 537 (496–583) to 392 (353–432) per 100 000, with an AAPC of −1.01% (−1.08% to −0.94%) (Figure 1 top).

Joinpoint regression analysis revealed that while ASDR and ASMR consistently decreased, both ASIR and ASPR showed fluctuations. ASIR had joinpoints in 1995, 2004, and 2013, and ASPR in 1995 and 2003. Prevalence increased from 1990 to 1995, declined from 1995 to 2003, and rose again after 2003.

The ASIR increase from 1990 to 1995 was mainly driven by lip and oral cavity cancer and other pharynx cancers (APC: 0.82% and 1.25%, Figure S1). Although all HNC types declined between 1994 and 2004, this trend reversed after 2013, with only larynx cancer continuing to decrease (−1.01%).

ASPR analysis indicated that nasopharynx, lip and oral cavity, and other pharynx cancers increased from 1990 to 1995 (APC: 0.66%, 0.85%, and 3.11%). The subsequent decline was driven by larynx and nasopharynx cancers, with the overall trend rising again after 2003 (Figure S2).

### 3.2 Differences in Gender

From 1990 to 2021, global ASPR, ASMR, ASDR, and ASIR for males aged 40–64 generally declined, with AAPCs of −0.09%, −1.04%, −1.04%, and −0.39%, respectively. In females, ASMR and ASDR decreased (AAPC: −0.80%), ASPR increased (−0.63%), and ASIR remained largely unchanged (0.1%).

Joinpoint regression analysis revealed a monotonic decline in ASDR and ASMR among males. Both ASIR and ASPR increased from 1990 to 1995 (APC: 0.27%, 0.49%), then decreased (−1.31%, −0.89%), with a turning point in 2004. After this, ASIR continued to decline slowly (−0.10%), while ASPR rose (2004–2007: 0.69%; 2007–2021: 0.06%).

For females, ASMR and ASDR experienced three phases: an initial decline, a subsequent increase, and another decline, with turning points in 2012 and 2018. ASPR began rising from its lowest point in 1990 (0.76%), declined again after 1997 (−0.43%), and then consistently increased after 2003 (0.80%, 1.65%, and 0.15%). The ASIR trend mirrored that of ASPR, but its lowest point occurred in 2009.

### 3.3 Age‐Specific Global Trends

In the age groups divided by 5‐year increments, all HNC indicators showed a consistent upward trend with increasing age. Age‐specific trends in ASPR, ASIR, ASMR, and ASDR are presented in Figure S3.

### 3.4 Trends by SDI

**FIGURE 2:**
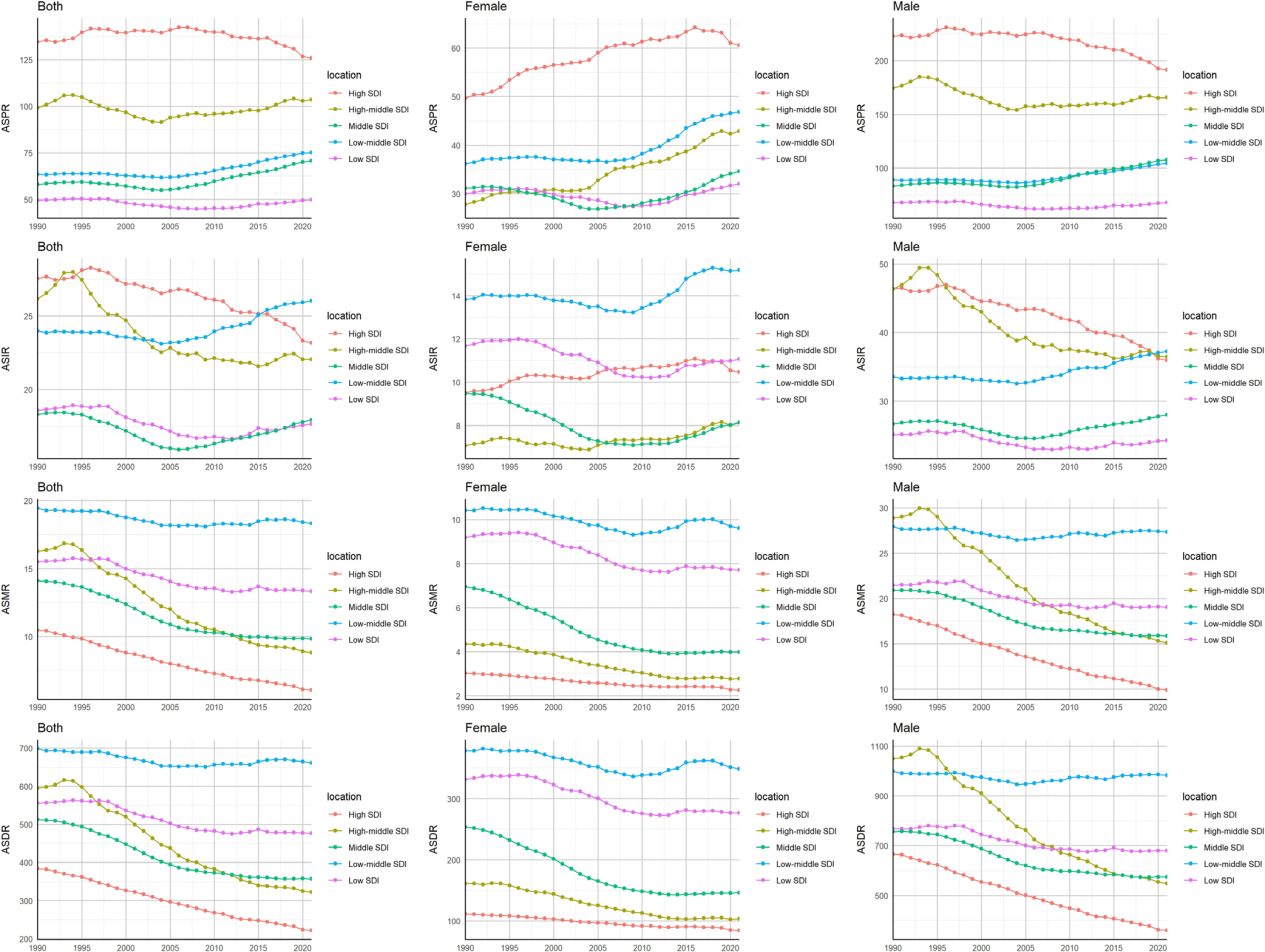
Trends in age‐standardized prevalence rate (ASPR), incidence rate (ASIR), mortality rate (ASMR), and DALYs rate (ASDR) of head and neck cancer (HNC) from 1990 to 2021, stratified by gender and sociodemographic index (SDI) region.

From 1990 to 2021, high SDI countries had the highest ASPR (134–126 per 100 000, Figure 2). Notable changes were seen in middle and low‐middle SDI countries (AAPCs: 0.64% and 0.58%, Table S1), driven by gender differences. In high SDI countries, female AAPC rose by 0.63%, while male AAPC fell by −0.5%. High‐middle SDI countries saw a female AAPC increase of 1.4%, contrasting with a male decrease of −0.19%. Middle and low‐middle SDI countries showed rising AAPCs for both genders.

ASIR trends declined in high and high‐middle SDI countries, driven by male ASIR decreases (−0.81% and −0.72%, respectively). In low‐middle SDI countries, ASIR increased, largely due to male ASIR (33.5–37.2 per 100 000).

ASMR and ASDR declined across all SDI groups, especially in high‐middle SDI countries (overall: −1.93%, males: −2.05%).

### 3.5 Trends Across 204 Countries and Territories

From 1990 to 2021, Cabo Verde saw the largest increases in HNC prevalence, incidence, mortality, and DALYs among individuals aged 40–64, with AAPCs around 4%–5%. Conversely, Kuwait experienced the steepest declines across all indicators (AAPCs: −3.66% to −5.02%).

France initially had the highest prevalence and incidence of HNC, but by 2021, Taiwan took the lead. Pakistan had the highest ASMR and ASDR by 2021, particularly among women. Detailed country‐level ASRs and their trends from 1990 to 2021 are summarized in Table S3.

In Southeast Asia, Vietnam had the highest increase in incidence (AAPC: 0.91%) and ASR (31.01 per 100 000) by 2021, while Singapore had the fastest decline (−2.08%). Taiwan showed the most significant incidence increase in East Asia (AAPC: 2.05%, ASR: 74.41 per 100 000), whereas Mongolia had the fastest decline.

**FIGURE 3:**
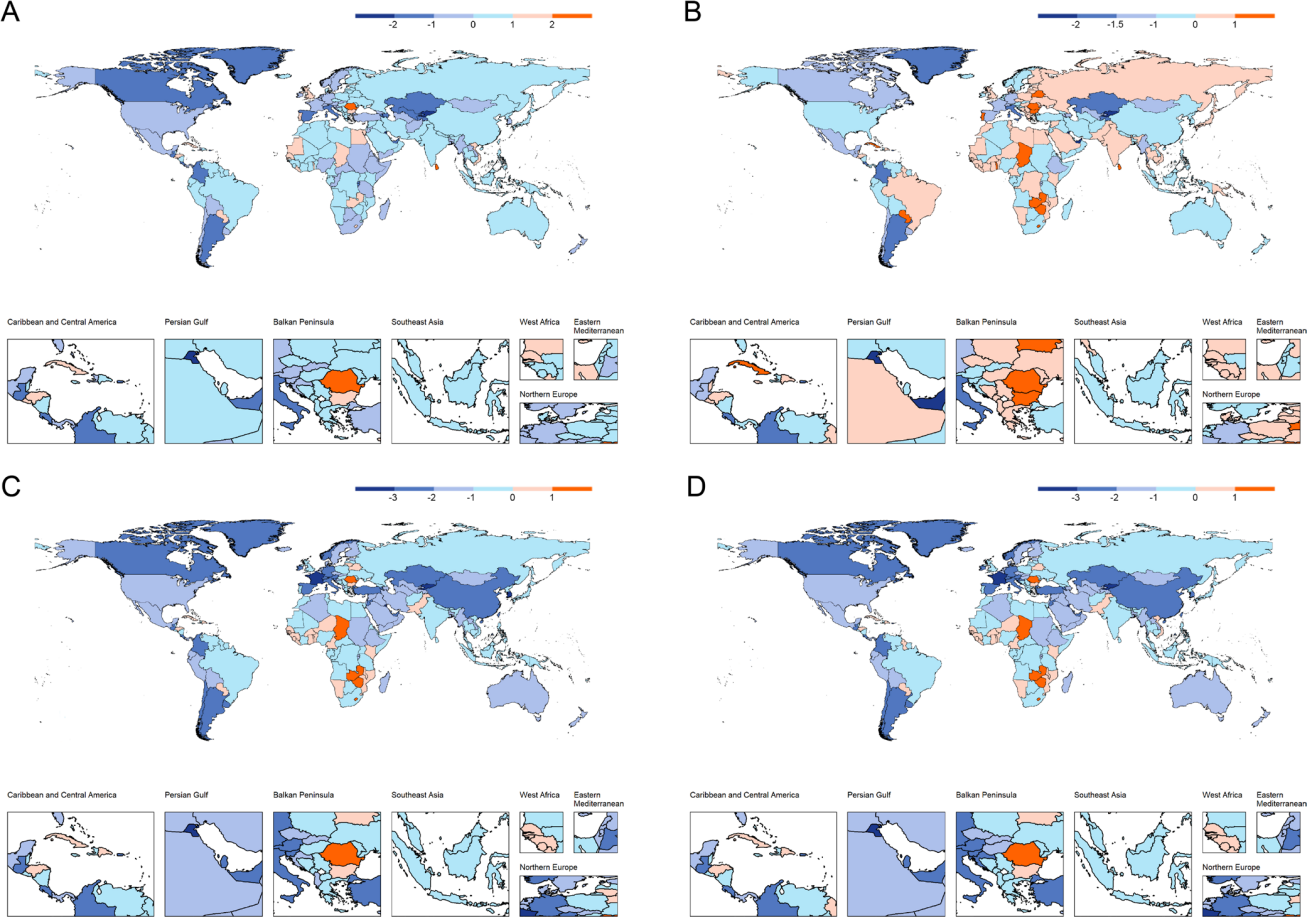
Global geographical distribution of the average annual percentage change (AAPC) in head and neck cancer (HNC) burden between 1990 and 2021. Subpanels show AAPC for: (A) age‐standardized prevalence rate (ASPR), (B) incidence rate (ASIR), (C) mortality rate (ASMR), and (D) DALYs rate (ASDR). Positive values (in shades of red) indicate increasing trends, while negative values (in shades of blue) reflect decreasing trends. Countries with deeper colors experienced more pronounced changes.

In Latin America, Cuba had the highest incidence increase (1.51%), while Argentina saw the steepest decline (−2.24%) (Figure 3).

### 3.6 Predictive Analysis on HNC Burden to 2045

**FIGURE 4:**
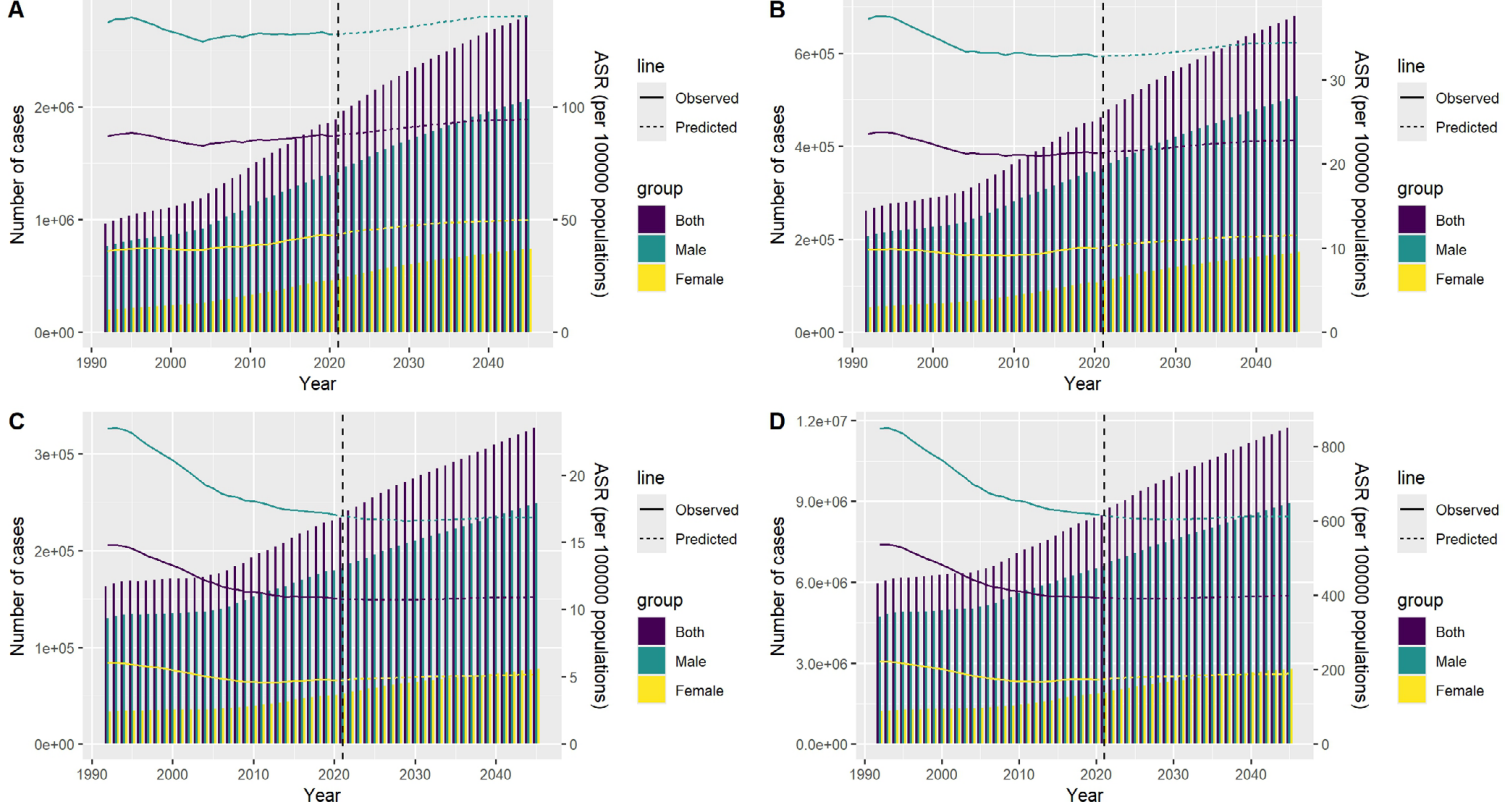
Projected global trends in head and neck cancer (HNC) burden from 2022 to 2045 using the Nordpred age–period–cohort model. Subpanels depict predicted changes in: (A) prevalence, (B) incidence, (C) mortality, and (D) DALYs, showing both the absolute number of cases and age‐standardized rates (ASRs). The projections highlight anticipated increases in prevalence and incidence, while mortality and DALY rates are expected to remain stable.

The predicted case number and ASR of incidence, prevalence, and DALYs of HNC to 2045 were illustrated in Figure 4. Overall, an increase in prevalence, incidence, mortality, and DALYs was predicted. ASR of prevalence and incidence was predicted to increase slowly, while mortality and DALYs were stable. For detailed values, see Table S2.

### 3.7 Attributable Risk Factors

**FIGURE 5:**
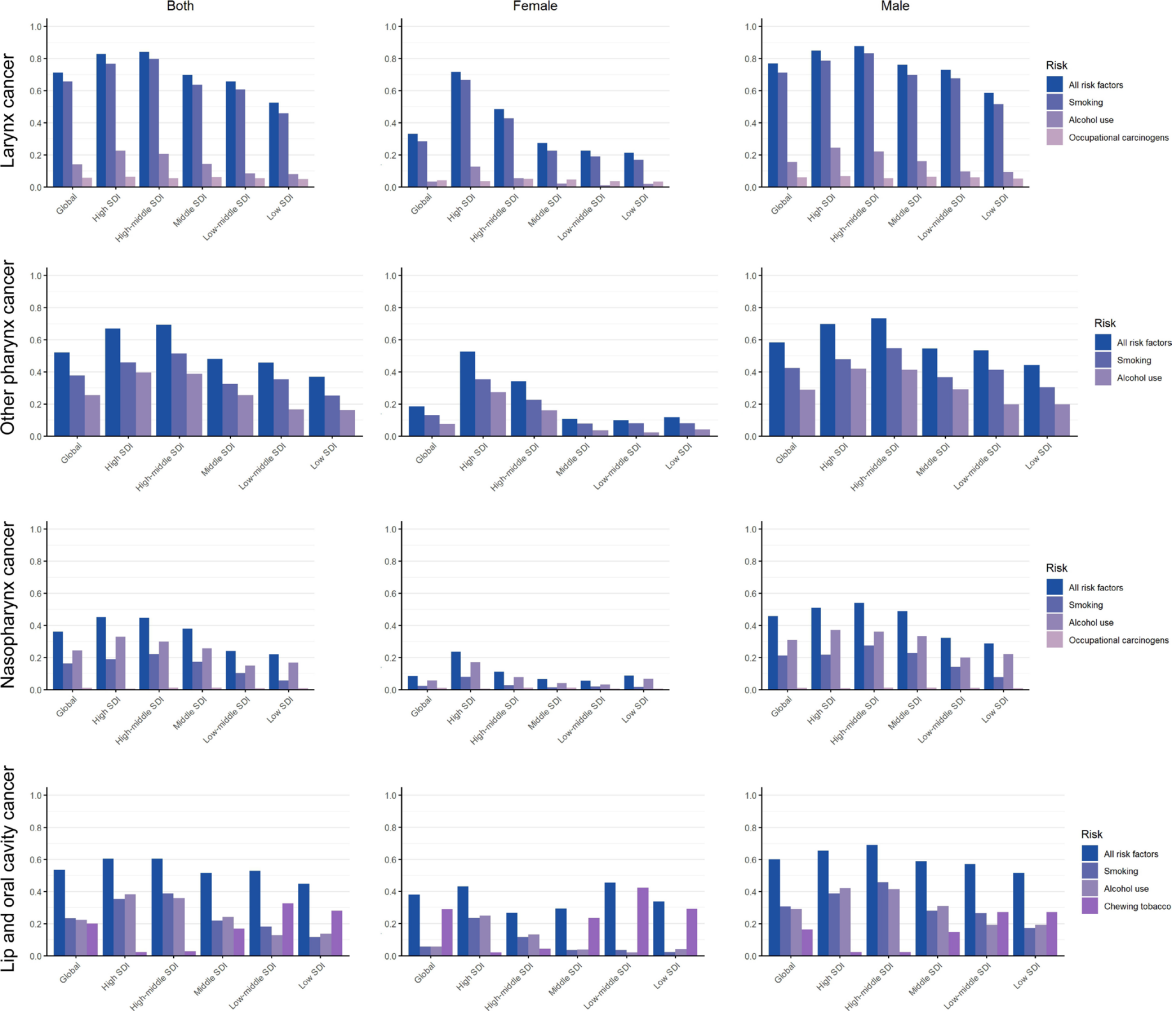
Population attributable fraction (PAF) of disability‐adjusted life years (DALYs) for head and neck cancer (HNC) in 2021, attributed to major risk factors including smoking, alcohol use, occupational carcinogens, and chewing tobacco. Results are presented globally and stratified by sex.

The population attributable fraction (PAF) estimates how much disease incidence could decrease if specific exposures were eliminated. A reanalysis of PAF data from 1990 to 2021 identified smoking, alcohol use, occupational carcinogens, and chewing tobacco as the primary risk factors for DALYs related to HNC (Figure 5). Smoking and alcohol were the leading risks for larynx and other pharynx cancers, with occupational carcinogens being the second most significant for middle‐aged women in middle and low SDI countries. In nasopharynx cancer, alcohol, smoking, and occupational carcinogens were key risks, with higher PAFs in males across all SDI levels. Chewing tobacco was a significant risk for lip and oral cavity cancer, especially among women in middle and low SDI regions. Overall, PAFs were higher in high and high‐middle SDI regions, with males generally showing higher PAFs than females.

## 4 Discussion

The purpose of this study was to evaluate the global burden of HNCs in adults aged 40–64 from 1990 to 2021. To the best of our knowledge, this study is the first to comprehensively analyze the burden, trends, and gender differences of middle‐aged HNC at both global and national levels. This age group represents a critical period for the onset of HNCs, while also playing significant societal roles and exerting substantial influence within families. Therefore, research on this demographic holds profound implications for the sustainable economic development of individuals, families, and society. Although their lifestyle habits may have already been established, they still exhibit considerable plasticity, making interventions during this stage potentially more acceptable and actionable. The primary risk factors for HNCs often become apparent in individuals aged 40–64; however, these risk factors remain modifiable to a significant extent. Based on the findings of this study, region‐specific, targeted health guidance could effectively reduce the incidence and burden of HNCs and related diseases.

Our analysis showed that while the prevalence of HNC in all groups has nearly doubled, the ASPR has remained relatively stable. In contrast, the ASIR, ASMR, and ASDR have all shown a decreasing trend.

The findings of increasing prevalence yet stable or declining ASR trends align with previous studies that have noted similar patterns globally [20]. This can be attributed to advancements in diagnostic techniques leading to better detection of cases, particularly in countries with high SDI scores [21, 22, 23]. However, the declining mortality and DALY rates indicate improvements in treatment outcomes and possibly early detection in many regions [24]. This may explain why, in the joinpoint regression analysis, ASIR remained stable after 2003 while ASPR increased and ASMR decreased.

Our analysis revealed notable gender differences in HNC trends. Males generally exhibited higher ASIR, ASMR, and ASDR compared to females across the study period. However, it is noteworthy that in some regions, particularly in lower SDI countries, the rate of increase in HNC prevalence and incidence was more pronounced among females. This could be attributed to changing lifestyle factors such as increased tobacco and alcohol consumption among women [25], as well as occupational exposure to carcinogens, which have traditionally been higher in males.

For lip and oral cavity cancer and other pharynx cancer, smoking is the predominant risk factor across all SDI levels and genders, but it is particularly pronounced in males, especially in high and high‐middle SDI countries. Among females, the PAF for smoking is lower, while other factors such as chewing tobacco are relatively more significant in low and low‐middle SDI regions. This suggests that while tobacco‐related interventions have been effective in reducing male cancer burdens in higher SDI regions, different integrated strategies might be needed for females [26], particularly in regions with lower socioeconomic development.

In the case of larynx cancer, smoking remains the leading risk factor globally, with higher PAF in males than females [27]. Occupational carcinogens also contribute significantly, particularly in lower SDI regions, where males are more likely to be exposed due to occupational hazards. For females, although smoking is still a significant risk, the overall PAF is lower, indicating the need for targeted occupational safety measures in addition to smoking cessation programs.

The lower PAF observed for nasopharyngeal cancer (NPC) in the initial analysis is primarily due to the exclusion of Epstein–Barr virus (EBV) data in the GBD study. EBV is a significant risk factor, particularly in endemic regions like Southern China and Southeast Asia, where it accounts for up to 90% of NPC cases [28]. The absence of EBV‐specific data leads to an underestimation of NPC's actual burden in these areas. In another study, EBV‐attributable proportions for specific cancer types were first determined based on published literature and subsequently applied to GBD estimates to calculate incidence, mortality, and DALYs. Collectively, EBV‐associated malignancies accounted for an estimated 4.6 million DALYs globally in 2017. Among these, NPC contributed approximately 44.5% of the total EBV‐related DALY burden [29]. This revised understanding highlights the importance of including EBV in the assessment of NPC burden. Public health strategies should focus not only on traditional risk factors like smoking and alcohol but also on mitigating EBV infection, including the potential development of EBV vaccines or other preventive measures, particularly in high‐risk regions. For example, Taiwan may need to formulate special policies for the cultivation and consumption of betel nut [30], and learn from the experience of clinics in areas where interventions have been effective, such as Singapore [31]. Another limitation of our study is that the GBD database does not distinguish HPV‐related from unrelated subtypes of HNC, particularly for oropharyngeal cancers, which are increasingly driven by HPV infection. As a result, etiological shifts cannot be fully captured. The rising incidence of oropharyngeal cancer in high‐ and middle‐SDI countries likely reflects the growing burden of HPV‐associated cases [32], which differ markedly from HPV‐negative tumors in terms of etiology, prognosis, and treatment response [3]. As their proportion continues to increase, they may significantly alter future disease patterns. Moreover, expanding HPV vaccination programs—especially among adolescents—could reduce future incidence. Recent modeling work has projected that implementing gender‐neutral HPV vaccination strategies could significantly reduce the long‐term incidence of HPV‐related oropharyngeal cancer, particularly among men [33]. This highlights the importance of expanding vaccine coverage and enhancing subtype‐specific cancer surveillance.

Considering the gender differences observed in HNC, it is imperative that public health strategies are adapted to address the specific needs and risk profiles of men and women. Males consistently show higher rates of incidence, mortality, and DALYs, which suggests that they are at greater risk, potentially due to higher exposure to risk factors like smoking, alcohol consumption, and occupational hazards. Public health interventions must therefore intensify efforts to reduce these risk factors among males, particularly in lower SDI regions where these behaviors are more prevalent [12].

For females, the rising trends of tobacco and alcohol use—particularly in lower SDI regions—highlight an increasing burden of HNC that calls for targeted interventions, including public education and risk reduction strategies. In addition to behavioral factors, part of the observed increase may also be attributed to improved diagnostic capacity and healthcare access, which could enhance case detection and partially explain the upward trends.

This study, based on GBD 2021 data, projects the global burden of HNC from 2022 to 2045, predicting slight increases in ASPR and ASIR, with ASMR and ASDR remaining stable. These projections indicate that HNC will continue to pose a significant public health challenge. Beyond established risk factors like smoking, alcohol consumption, and EBV infection, emerging environmental factors and lifestyle changes, such as urbanization, air pollution, and dietary shifts, are likely to influence HNC incidence, especially in rapidly developing regions. The integration of personalized medicine—tailoring prevention and treatment to individual genetic, environmental, and lifestyle factors—alongside efforts to address global healthcare disparities, will be essential in reducing the global HNC burden in the coming decades.

## 5 Conclusion

This study highlights the global burden of HNC among adults aged 40–64 from 1990 to 2021. While the overall prevalence has remained stable, incidence, mortality, and DALYs have decreased. Significant gender differences were identified, with males having higher rates, though a faster rise in female cases is seen in lower SDI regions. These findings underscore the need for gender‐ and region‐specific strategies to address HNC risk factors and improve prevention efforts globally.

## Author Contributions

**Zhuoding Luo:** conceptualization (equal), investigation (equal), methodology (lead), software (lead), writing – original draft (lead), writing – review and editing (equal). **Yihan Huang:** data curation (equal), formal analysis (equal), writing – original draft (equal). **Renjing Ye:** data curation (equal), formal analysis (equal), writing – original draft (equal). **Min Yin:** conceptualization (lead), project administration (lead), supervision (lead), writing – review and editing (lead).

## Ethics Statement

The authors have nothing to report.

## Consent

The authors have nothing to report.

## Conflicts of Interest

The authors declare no conflicts of interest.

## Supporting information


**Data S1.** Supporting information.

## Data Availability

The data that support the findings of this study are available from the corresponding author upon reasonable request.
