# Supplementary material for: Global Burden and Gender Disparities in Head and Neck Cancers Among Adults Aged 40–64, 1990–2021: A Systematic Analysis From the Global Burden of Disease Study 2021
Source: Cancer Rep (Hoboken). 2025 Aug 20;8(8):e70287. doi: 10.1002/cnr2.70287 (PMC12365661; doi:10.1002/cnr2.70287)
Supplement: Supplementary file 1 — Data S1. Supporting information. [file CNR2-8-e70287-s001.docx]

**Supplementary Materials**

[**Figure 1**](#eFigure1) Joinpoint regression analysis of age-standardized prevalence rate (ASPR) and age-standardized incidence rate (ASIR) in global for larynx cancer, nasopharynx cancer, other pharynx cancers, and lip and oral cavity cancer.

[**Figure 2**](#eFigure2) Stacked bar charts of ASDR, ASMR, ASIR and ASPR in global for larynx cancer, nasopharynx cancer, other pharynx cancers, and lip and oral cavity cancer.

[**Figure 3**](#eFigure3) Trend in ASDR, ASMR, ASIR and ASPR across different age groups.

[**Table 1**](#eTable1) Data of age-standardized prevalence, incidence, mortality and DALYs rates and AAPC of HNC at global and regional level, 1990 to 2021.

[**Table 2**](#eTable21) Nordpred projections of case numbers and age-standardized rate (ASR), up to the year 2045.

[age-standardized prevalence rate (ASPR)](#eTable21)

[age-standardized incidence rate (ASIR)](#eTable22)

[age-standardized mortality rate (ASMR)](#eTable23)

[age-standardized DALYs rate (ASDR)](#eTable24)

[**Table 3**](#eTable3) The age-standardized rates and AAPCs of Head and Neck Cancers Among Adults Aged 40-64 in 204 Countries and Territories.

Supplementary Figure 1. Joinpoint regression analysis of age-standardized prevalence rate (ASPR) and age-standardized incidence rate (ASIR) in global for larynx cancer, nasopharynx cancer, other pharynx cancers, and lip and oral cavity cancer.


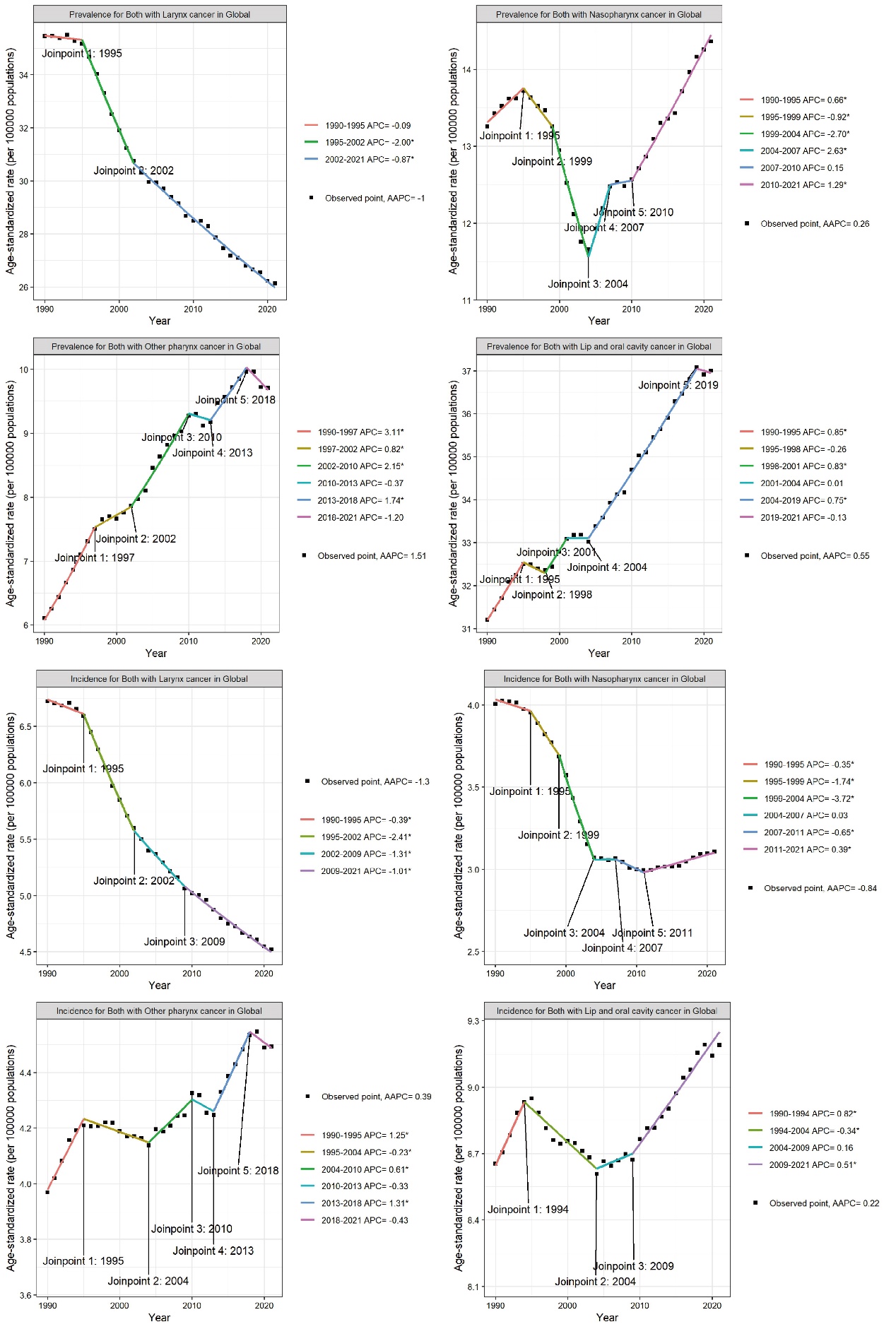


Supplementary Figure 2. Stacked bar charts of ASDR, ASMR, ASIR and ASPR in global for larynx cancer, nasopharynx cancer, other pharynx cancers, and lip and oral cavity cancer.


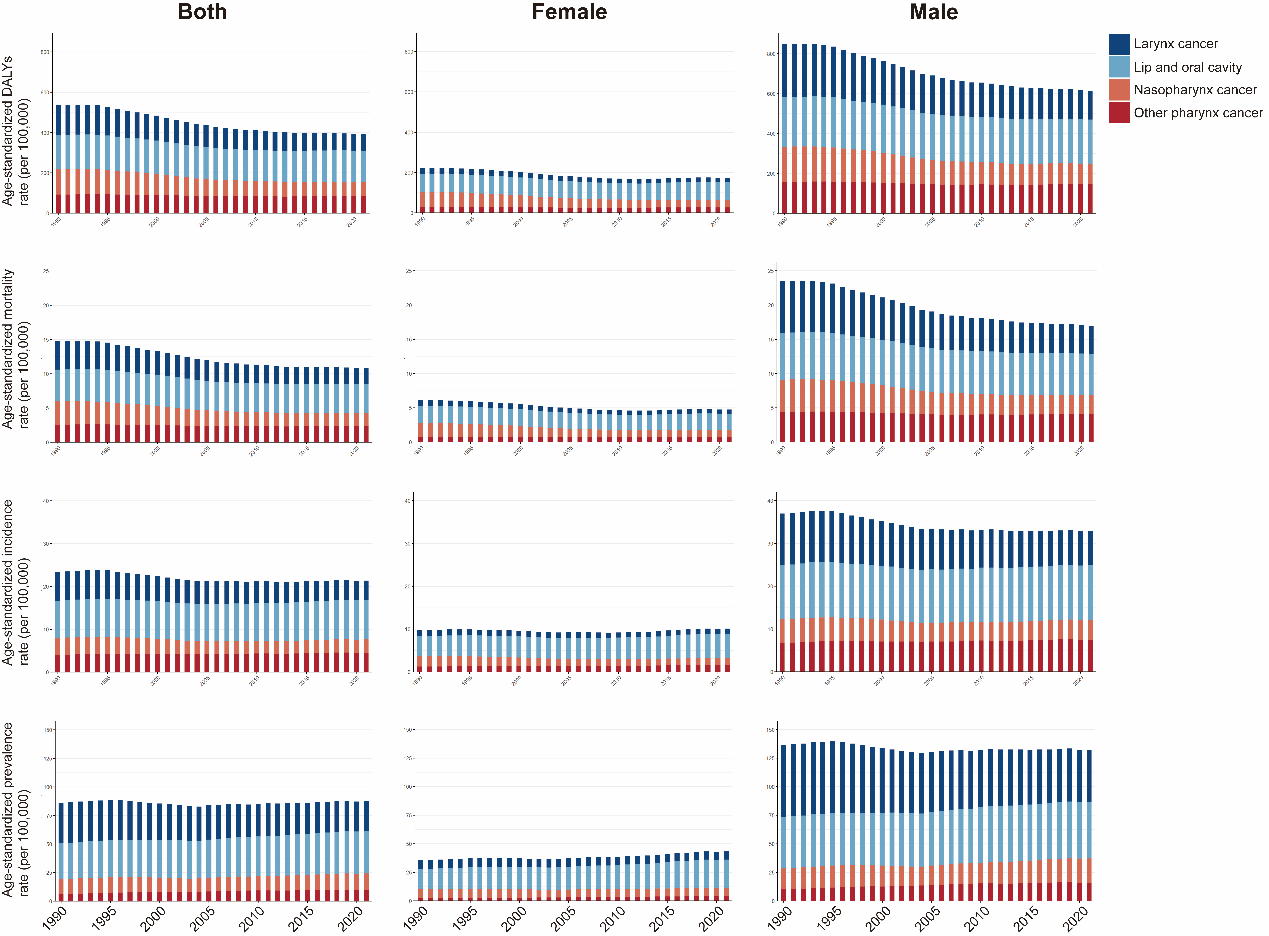


Supplementary Figure 3. Trend in ASDR, ASMR, ASIR and ASPR across different age groups.


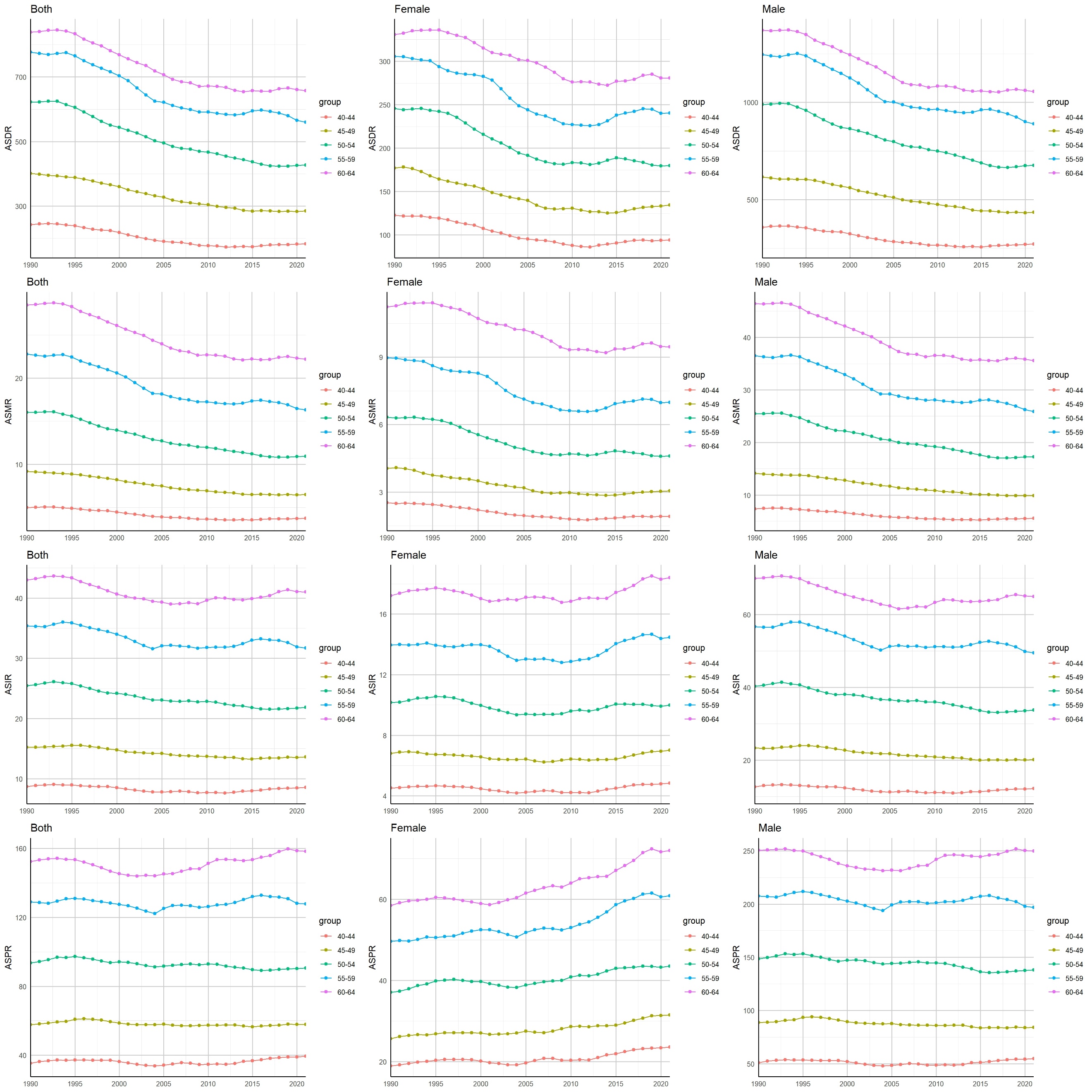


Table 1. Data of age-standardized prevalence, incidence, mortality and DALYs rates and AAPC of HNC at global and regional level, 1990 to 2021.

|  | Prevalence (95% UI) | | | |  | Incidence (95% UI) | | | |  | Deaths | | | |  | DALYs | | | |  |
| --- | --- | --- | --- | --- | --- | --- | --- | --- | --- | --- | --- | --- | --- | --- | --- | --- | --- | --- | --- | --- |
|  | cases in 1990 (000s) | ASR in 1990 (per 100,000) | cases in 2021 (000s) | ASR in 2021 (per 100,000) | AAPC (95% CI) | cases in 1990 (000s) | ASR in 1990 (per 100,000) | cases in 2021 (000s) | ASR in 2021 (per 100,000) | AAPC (95% CI) | cases in 1990 (000s) | ASR in 1990 (per 100,000) | cases in 2021 (000s) | ASR in 2021 (per 100,000) | AAPC (95% CI) | cases in 1990 (000s) | ASR in 1990 (per 100,000) | cases in 2021 (000s) | ASR in 2021 (per 100,000) | AAPC (95% CI) |
| **Global** | 918(866 to 974) | 86.0(81.2 to 91.3) | 1887(1725 to 2067) | 87.2(79.7 to 95.5) | 0.07 (0.01 to 0.12) | 249(233 to 266) | 23.4(21.9 to 25.0) | 462(421 to 505) | 21.3(19.4 to 23.3) | -0.28 (-0.33 to -0.23) | 157(145 to 171) | 14.8(13.6 to 16.0) | 234(211 to 257) | 10.8(9.73 to 11.8) | -1.01 (-1.09 to -0.92) | 5734(5290 to 6219) | 537(496 to 583) | 8489(7646 to 9338) | 392(353 to 432) | -1.01 (-1.08 to -0.94) |
| **Sex:** |  |  |  |  |  |  |  |  |  |  |  |  |  |  |  |  |  |  |  |  |
| Female | 188(169 to 206) | 35.4(31.6 to 38.8) | 471(420 to 532) | 43.2(38.5 to 48.8) | 0.63 (0.51 to 0.75) | 51.8(45.5 to 57.8) | 9.73(8.56 to 10.9) | 110(97.4 to 126) | 10.1(8.91 to 11.5) | 0.10(0 to 0.20) | 32.2(27.5 to 36.8) | 6.07(5.17 to 6.92) | 51.8(45.1 to 60.3) | 4.73(4.11 to 5.50) | -0.80(-0.92 to -0.69) | 1185(1009 to 1353) | 223(190 to 255) | 1897(1649 to 2206) | 174(151 to 202) | -0.80 (-0.91 to -0.70) |
| Male | 729(682 to 782) | 136(128 to 146) | 1417(1268 to 1580) | 132(118 to 148) | -0.09 (-0.15 to -0.02) | 197(183 to 213) | 36.9(34.3 to 39.9) | 352(314 to 390) | 32.8(29.3 to 36.4) | -0.39 (-0.43 to -0.35) | 125(114 to 138) | 23.5(21.4 to 25.8) | 182(161 to 203) | 17.0(15.0 to 19.0) | -1.04(-1.10 to -0.97) | 4549(4147 to 5007) | 850(775 to 935) | 6592(5820 to 7383) | 615(543 to 689) | -1.04 (-1.10 to -0.98) |
| **Age group(years):** |  |  |  |  |  |  |  |  |  |  |  |  |  |  |  |  |  |  |  |  |
| 40-44 | 101(94.1 to 109) | 35.3(32.8 to 38.0) | 196(174 to 222) | 39.4(34.7 to 44.3) | 0.39 (0.16 to 0.63) | 24.9(23.1 to 27.0) | 8.71(8.05 to 9.41) | 43.0(37.9 to 48.0) | 8.59(7.58 to 9.59) | -0.02 (-0.21 to 0.17) | 14.3(13.0 to 15.7) | 4.99(4.53 to 5.46) | 18.8(16.6 to 21.0) | 3.75(3.31 to 4.19) | -0.92 (-1.08 to -0.76) | 695(632 to 760) | 242(221 to 265) | 918(809 to 1024) | 184(162 to 205) | -0.90 (-1.06 to -0.74) |
| 45-49 | 134(126 to 143) | 57.7(54.3 to 61.5) | 274(246 to 305) | 57.9(51.9 to 64.5) | 0.04 (-0.11 to 0.19) | 35.4(33.0 to 37.9) | 15.2(14.2 to 16.3) | 64.6(57.8 to 71.6) | 13.6(12.2 to 15.1) | -0.34 (-0.42 to -0.26) | 21.3(19.7 to 23.3) | 9.19(8.47 to 10.0) | 30.8(27.2 to 34.4) | 6.50(5.74 to 7.25) | -1.12 (-1.19 to -1.05) | 931(858 to 1015) | 401(370 to 437) | 1350(1193 to 1503) | 285(252 to 318) | -1.10 (-1.17 to -1.04) |
| 50-54 | 198(187 to 211) | 93.5(88.2 to 99.2) | 403(366 to 446) | 90.6(82.3 to 100) | -0.10(-0.21 to 0.01) | 54.0(50.5 to 57.8) | 25.4(23.8 to 27.2) | 97.3(88.2 to 107) | 21.9(19.8 to 24.1) | -0.49 (-0.62 to -0.37) | 34.0(31.4 to 36.9) | 16.0(14.8 to 17.3) | 48.7(43.7 to 53.8) | 10.9(9.81 to 12.1) | -1.23 (-1.37 to -1.09) | 1323(1218 to 1430) | 622(573 to 673) | 1901(1703 to 2100) | 427(383 to 472) | -1.21 (-1.34 to -1.09) |
| 55-59 | 238(226 to 253) | 129(122 to 137) | 506(468 to 550) | 128(118 to 139) | 0.00(-0.17 to 0.17) | 65.5(61.4 to 70.0) | 35.4(33.1 to 37.8) | 126(116 to 136) | 31.7(29.3 to 34.4) | -0.32 (-0.5 to -0.15) | 42.1(39.0 to 45.7) | 22.8(21.0 to 24.7) | 64.5(58.9 to 70.5) | 16.3(14.9 to 17.8) | -1.04(-1.20 to -0.89) | 1439(1331 to 1560) | 777(719 to 842) | 2216(2019 to 2422) | 560(510 to 612) | -1.02 (-1.19 to -0.86) |
| 60-64 | 244(233 to 258) | 152(145 to 161) | 507(472 to 544) | 158(148 to 170) | 0.14 (-0.03 to 0.32) | 69.1(65.1 to 73.5) | 43.0(40.5 to 45.8) | 131(121 to 141) | 41.0(37.9 to 44.2) | -0.12 (-0.29 to 0.05) | 45.7(42.4 to 49.3) | 28.4(26.4 to 30.7) | 70.9(64.8 to 77.2) | 22.2(20.3 to 24.1) | -0.77 (-0.86 to -0.68) | 1348(1251 to 1454) | 839(779 to 905) | 2104(1992 to 2288) | 657(601 to 715) | -0.76 (-0.84 to -0.68) |
| **SDI level:** |  |  |  |  |  |  |  |  |  |  |  |  |  |  |  |  |  |  |  |  |
| High | 329(312 to 349) | 134(127 to 142) | 498(470 to 529) | 126(119 to 134) | -0.23 (-0.46 to 0) | 67.5(63.8 to 71.7) | 27.5(26.1 to 29.3) | 92.3(87.0 to 98.2) | 23.2(21.8 to 24.7) | -0.54 (-0.79 to -0.28) | 25.7(24.3 to 27.2) | 10.5(9.89 to 11.1) | 24.4(23.0 to 25.9) | 6.05(5.69 to 6.43) | -1.77 (-1.91 to -1.62) | 935(883 to 991) | 384(362 to 406) | 875(821 to 932) | 222(208 to 236) | -1.78 (-1.89 to -1.67) |
| High-middle | 259(239 to 283) | 99.1(91.2 to 108) | 483(414 to 567) | 103(88.6 to 122) | 0.17 (0 to 0.34) | 68.6(63.2 to 74.9) | 26.2(24.1 to 28.6) | 104(90.5 to 120) | 22.1(19.2 to 25.5) | -0.50(-0.83 to -0.16) | 42.8(39.1 to 47.1) | 16.3(14.9 to 17.9) | 41.9(36.9 to 47.8) | 8.81(7.75 to 10.1) | -1.93(-2.22 to -1.64) | 1547(1414 to 1707) | 595(543 to 657) | 1515(1331 to 1733) | 322(283 to 369) | -1.93 (-2.21 to -1.66) |
| Middle | 177(159 to 197) | 57.9(51.9 to 64.3) | 517(450 to 593) | 70.7(61.6 to 81.1) | 0.64 (0.52 to 0.75) | 56.0(50.2 to 62.2) | 18.3(16.4 to 20.3) | 131(115 to 149) | 17.9(15.7 to 20.4) | -0.07 (-0.15 to 0.01) | 42.9(38.3 to 47.9) | 14.1(12.6 to 15.7) | 72.0(63.3 to 81.4) | 9.84(8.65 to 11.1) | -1.17 (-1.24 to -1.1) | 1574(1405 to 1757) | 512(457 to 571) | 2618(2298 to 2962) | 357(314 to 404) | -1.18 (-1.25 to -1.1) |
| Low-middle | 117(100 to 136) | 63.4(54.5 to 73.9) | 309(268 to 354) | 75.2(65.2 to 86.1) | 0.58 (0.46 to 0.69) | 43.9(37.4 to 51.6) | 24.0(20.4 to 28.2) | 107(91.4 to 123) | 26.0(22.4 to 30.0) | 0.27 (0.18 to 0.36) | 35.4(30.1 to 41.8) | 19.4(16.5 to 23.0) | 74.6(63.8 to 86.3) | 18.3(15.7 to 21.2) | -0.17 (-0.25 to -0.09) | 1288(1093 to 1522) | 698(593 to 825) | 2720(2320 to 3153) | 661(564 to 766) | -0.16 (-0.23 to -0.08) |
| Low | 34.0(27.3 to 41.3) | 49.5(39.9 to 60.1) | 78.3(63.9 to 94.6) | 49.9(40.8 to 60.1) | 0.02 (-0.12 to 0.15) | 12.7(10.2 to 15.5) | 18.6(14.9 to 22.7) | 27.4(22.2 to 33.4) | 17.7(14.3 to 21.4) | -0.17 (-0.28 to -0.05) | 10.6(8.46 to 13.0) | 15.5(12.4 to 19.0) | 20.5(16.6 to 25.1) | 13.3(10.8 to 16.3) | -0.49 (-0.64 to -0.35) | 383(307 to 470) | 556(445 to 682) | 753(606 to 924) | 477(385 to 584) | -0.50(-0.63 to -0.37) |

Table 2.1. Nordpred projections of case numbers and age-standardized prevalence rate (ASPR) up to the year 2045.

| **Prevalence** | Case number | | |  | ASR (per 100,000) | | |
| --- | --- | --- | --- | --- | --- | --- | --- |
| Year | Both | Female | Male |  | Both | Female | Male |
| 1992 | 963997 | 199756 | 764241 |  | 87.032477 | 36.111463 | 137.646556 |
| 1993 | 990682 | 205637 | 785045 |  | 87.864971 | 36.473156 | 138.95893 |
| 1994 | 1010252 | 211031 | 799221 |  | 88.018867 | 36.723097 | 139.049248 |
| 1995 | 1033425 | 217030 | 816395 |  | 88.480798 | 37.052547 | 139.704477 |
| 1996 | 1049009 | 222560 | 826449 |  | 88.100896 | 37.231231 | 138.837938 |
| 1997 | 1063089 | 227480 | 835609 |  | 87.437402 | 37.24424 | 137.57549 |
| 1998 | 1077386 | 232425 | 844961 |  | 86.794965 | 37.267961 | 136.305762 |
| 1999 | 1089615 | 237351 | 852264 |  | 85.904994 | 37.244511 | 134.589013 |
| 2000 | 1104645 | 241672 | 862972 |  | 85.299777 | 37.135127 | 133.5297 |
| 2001 | 1120583 | 245165 | 875419 |  | 84.593711 | 36.828651 | 132.462547 |
| 2002 | 1139217 | 250440 | 888777 |  | 83.907623 | 36.69601 | 131.250638 |
| 2003 | 1159728 | 255811 | 903917 |  | 83.247365 | 36.518129 | 130.125637 |
| 2004 | 1184848 | 263157 | 921691 |  | 82.726468 | 36.521876 | 129.106624 |
| 2005 | 1231936 | 275743 | 956194 |  | 83.689071 | 37.217283 | 130.368325 |
| 2006 | 1274874 | 286995 | 987879 |  | 84.114703 | 37.606969 | 130.884319 |
| 2007 | 1321959 | 298868 | 1023091 |  | 84.607708 | 37.967045 | 131.57648 |
| 2008 | 1365142 | 309632 | 1055510 |  | 84.747315 | 38.126456 | 131.797055 |
| 2009 | 1401587 | 319159 | 1082429 |  | 84.342556 | 38.075025 | 131.127105 |
| 2010 | 1456880 | 333913 | 1122966 |  | 85.021629 | 38.613958 | 132.059128 |
| 2011 | 1509124 | 347787 | 1161337 |  | 85.52617 | 39.049489 | 132.733324 |
| 2012 | 1545013 | 357344 | 1187669 |  | 85.368789 | 39.115019 | 132.426417 |
| 2013 | 1588161 | 371457 | 1216705 |  | 85.563556 | 39.642962 | 132.36163 |
| 2014 | 1629043 | 385146 | 1243897 |  | 85.868299 | 40.210772 | 132.467833 |
| 2015 | 1670588 | 401754 | 1268834 |  | 86.006676 | 40.982283 | 131.994397 |
| 2016 | 1714291 | 415870 | 1298421 |  | 86.527532 | 41.607054 | 132.429778 |
| 2017 | 1753998 | 429694 | 1324304 |  | 86.819604 | 42.186556 | 132.438138 |
| 2018 | 1798791 | 445094 | 1353696 |  | 87.381173 | 42.909735 | 132.844074 |
| 2019 | 1838738 | 456278 | 1382460 |  | 87.747248 | 43.231491 | 133.260083 |
| 2020 | 1855572 | 460750 | 1394821 |  | 87.102398 | 42.952944 | 132.259536 |
| 2021 | 1887263 | 470600 | 1416663 |  | 87.208286 | 43.177716 | 132.288242 |
| 2022 | 1964183 | 497274 | 1466909 |  | 88.065385 | 44.282265 | 132.822882 |
| 2023 | 2007457 | 511794 | 1495663 |  | 88.345918 | 44.744356 | 132.898755 |
| 2024 | 2052730 | 526993 | 1525737 |  | 88.628156 | 45.206447 | 132.974627 |
| 2025 | 2101394 | 541448 | 1559945 |  | 89.020904 | 45.576024 | 133.373071 |
| 2026 | 2148971 | 555758 | 1593213 |  | 89.411707 | 45.945602 | 133.771515 |
| 2027 | 2193948 | 569532 | 1624416 |  | 89.799224 | 46.31518 | 134.169959 |
| 2028 | 2235440 | 582538 | 1652901 |  | 90.184279 | 46.684758 | 134.568403 |
| 2029 | 2274127 | 594928 | 1679200 |  | 90.567998 | 47.054336 | 134.966847 |
| 2030 | 2312233 | 606131 | 1706102 |  | 90.983568 | 47.355378 | 135.505981 |
| 2031 | 2349262 | 617079 | 1732182 |  | 91.396923 | 47.65642 | 136.045115 |
| 2032 | 2386027 | 627957 | 1758070 |  | 91.807626 | 47.957462 | 136.584248 |
| 2033 | 2422060 | 638674 | 1783386 |  | 92.213302 | 48.258504 | 137.123382 |
| 2034 | 2456909 | 649128 | 1807782 |  | 92.614483 | 48.559546 | 137.662516 |
| 2035 | 2489001 | 657578 | 1831423 |  | 92.907313 | 48.717389 | 138.135016 |
| 2036 | 2521467 | 666070 | 1855398 |  | 93.202443 | 48.875232 | 138.607515 |
| 2037 | 2555141 | 674782 | 1880359 |  | 93.50285 | 49.033075 | 139.080015 |
| 2038 | 2590454 | 683817 | 1906637 |  | 93.807567 | 49.190918 | 139.552514 |
| 2039 | 2626884 | 693067 | 1933817 |  | 94.114478 | 49.34876 | 140.025014 |
| 2040 | 2656501 | 701316 | 1955185 |  | 94.178593 | 49.436334 | 140.066933 |
| 2041 | 2685969 | 709481 | 1976488 |  | 94.246884 | 49.523907 | 140.108853 |
| 2042 | 2714723 | 717424 | 1997299 |  | 94.32008 | 49.61148 | 140.150773 |
| 2043 | 2743678 | 725367 | 2018311 |  | 94.398477 | 49.699053 | 140.192692 |
| 2044 | 2773222 | 733394 | 2039828 |  | 94.481904 | 49.786627 | 140.234612 |
| 2045 | 2802003 | 741161 | 2060843 |  | 94.570237 | 49.8742 | 140.276532 |

Table 2.2. Nordpred projections of case numbers and age-standardized incidence rate (ASIR) up to the year 2045.

| **Incidence** | Case number | | |  | ASR (per 100,000) | | |
| --- | --- | --- | --- | --- | --- | --- | --- |
| Year | Both | Female | Male |  | Both | Female | Male |
| 1992 | 260759 | 54360 | 206399 |  | 23.570551 | 9.840765 | 37.224606 |
| 1993 | 267463 | 55622 | 211841 |  | 23.760716 | 9.885823 | 37.562878 |
| 1994 | 272081 | 56687 | 215395 |  | 23.754932 | 9.891378 | 37.554846 |
| 1995 | 276125 | 57774 | 218351 |  | 23.703021 | 9.89832 | 37.461824 |
| 1996 | 278153 | 58735 | 219418 |  | 23.430503 | 9.865937 | 36.969018 |
| 1997 | 280435 | 59659 | 220775 |  | 23.139843 | 9.809711 | 36.465211 |
| 1998 | 283145 | 60629 | 222515 |  | 22.888576 | 9.764059 | 36.019015 |
| 1999 | 285924 | 61502 | 224423 |  | 22.62073 | 9.693108 | 35.564515 |
| 2000 | 288631 | 62155 | 226476 |  | 22.366796 | 9.594787 | 35.165833 |
| 2001 | 291115 | 62660 | 228455 |  | 22.057468 | 9.45881 | 34.693228 |
| 2002 | 294486 | 63583 | 230903 |  | 21.770451 | 9.363672 | 34.220711 |
| 2003 | 298353 | 64503 | 233850 |  | 21.500261 | 9.258725 | 33.788361 |
| 2004 | 302629 | 65671 | 236958 |  | 21.216057 | 9.168301 | 33.31587 |
| 2005 | 312125 | 67976 | 244149 |  | 21.294759 | 9.23513 | 33.413081 |
| 2006 | 319615 | 69847 | 249768 |  | 21.179596 | 9.215823 | 33.214576 |
| 2007 | 329246 | 72104 | 257142 |  | 21.157087 | 9.218021 | 33.183617 |
| 2008 | 339440 | 74234 | 265206 |  | 21.147508 | 9.190303 | 33.218353 |
| 2009 | 347677 | 76211 | 271466 |  | 20.982605 | 9.129526 | 32.972089 |
| 2010 | 360888 | 79346 | 281543 |  | 21.110993 | 9.204998 | 33.182869 |
| 2011 | 372177 | 82251 | 289927 |  | 21.131606 | 9.256017 | 33.198257 |
| 2012 | 379988 | 84348 | 295641 |  | 21.029376 | 9.248386 | 33.019955 |
| 2013 | 389304 | 87406 | 301898 |  | 20.999712 | 9.337653 | 32.889714 |
| 2014 | 398902 | 90602 | 308300 |  | 21.047087 | 9.463832 | 32.875727 |
| 2015 | 410133 | 94742 | 315391 |  | 21.124961 | 9.661147 | 32.841095 |
| 2016 | 420340 | 97880 | 322459 |  | 21.220522 | 9.785035 | 32.912736 |
| 2017 | 429816 | 100992 | 328824 |  | 21.273966 | 9.903664 | 32.902529 |
| 2018 | 440581 | 104351 | 336230 |  | 21.394853 | 10.045267 | 33.005262 |
| 2019 | 449522 | 106681 | 342841 |  | 21.439208 | 10.090689 | 33.049763 |
| 2020 | 453598 | 107620 | 345978 |  | 21.27665 | 10.014157 | 32.803695 |
| 2021 | 461676 | 110172 | 351504 |  | 21.314443 | 10.08723 | 32.816596 |
| 2022 | 479292 | 116096 | 363196 |  | 21.471486 | 10.318811 | 32.879985 |
| 2023 | 489490 | 119400 | 370089 |  | 21.518203 | 10.415523 | 32.870552 |
| 2024 | 500198 | 122874 | 377324 |  | 21.565382 | 10.512234 | 32.86112 |
| 2025 | 511734 | 126180 | 385554 |  | 21.639986 | 10.588036 | 32.929445 |
| 2026 | 522966 | 129441 | 393525 |  | 21.714098 | 10.663838 | 32.99777 |
| 2027 | 533505 | 132559 | 400946 |  | 21.787366 | 10.73964 | 33.066095 |
| 2028 | 543122 | 135473 | 407649 |  | 21.860023 | 10.815441 | 33.13442 |
| 2029 | 552001 | 138224 | 413777 |  | 21.932363 | 10.891243 | 33.202745 |
| 2030 | 561029 | 140793 | 420236 |  | 22.024765 | 10.95868 | 33.321828 |
| 2031 | 569790 | 143302 | 426488 |  | 22.116567 | 11.026117 | 33.440911 |
| 2032 | 578498 | 145803 | 432695 |  | 22.20763 | 11.093554 | 33.559994 |
| 2033 | 587031 | 148273 | 438757 |  | 22.297349 | 11.160991 | 33.679077 |
| 2034 | 595267 | 150682 | 444585 |  | 22.385888 | 11.228428 | 33.79816 |
| 2035 | 602922 | 152641 | 450282 |  | 22.451699 | 11.263671 | 33.905718 |
| 2036 | 610679 | 154613 | 456066 |  | 22.51808 | 11.298914 | 34.013276 |
| 2037 | 618747 | 156643 | 462104 |  | 22.585794 | 11.334157 | 34.120834 |
| 2038 | 627229 | 158754 | 468475 |  | 22.654589 | 11.369401 | 34.228393 |
| 2039 | 635994 | 160921 | 475072 |  | 22.723921 | 11.404644 | 34.335951 |
| 2040 | 643340 | 162882 | 480458 |  | 22.741786 | 11.424901 | 34.352079 |
| 2041 | 650667 | 164830 | 485837 |  | 22.760699 | 11.445157 | 34.368208 |
| 2042 | 657838 | 166733 | 491105 |  | 22.780855 | 11.465414 | 34.384337 |
| 2043 | 665095 | 168650 | 496445 |  | 22.802321 | 11.485671 | 34.400466 |
| 2044 | 672532 | 170602 | 501930 |  | 22.82505 | 11.505928 | 34.416595 |
| 2045 | 679794 | 172497 | 507297 |  | 22.849023 | 11.526184 | 34.432723 |

Table 2.3. Nordpred projections of case numbers and age-standardized mortality rate (ASMR) up to the year 2045.

| **Mortality** | Case number | | |  | ASR (per 100,000) | | |
| --- | --- | --- | --- | --- | --- | --- | --- |
| Year | Both | Female | Male |  | Both | Female | Male |
| 1992 | 163225 | 33451 | 129773 |  | 14.770287 | 6.061294 | 23.440304 |
| 1993 | 166212 | 33956 | 132256 |  | 14.788128 | 6.043488 | 23.496693 |
| 1994 | 167904 | 34275 | 133629 |  | 14.689108 | 5.992453 | 23.356079 |
| 1995 | 168773 | 34517 | 134256 |  | 14.524887 | 5.929077 | 23.102735 |
| 1996 | 168429 | 34720 | 133709 |  | 14.232185 | 5.850994 | 22.608103 |
| 1997 | 168776 | 34966 | 133810 |  | 13.974253 | 5.769462 | 22.187668 |
| 1998 | 169397 | 35265 | 134132 |  | 13.744658 | 5.700346 | 21.804402 |
| 1999 | 170160 | 35464 | 134696 |  | 13.514561 | 5.6114 | 21.439106 |
| 2000 | 170818 | 35524 | 135293 |  | 13.292418 | 5.508047 | 21.104681 |
| 2001 | 170963 | 35528 | 135435 |  | 13.01238 | 5.389054 | 20.669245 |
| 2002 | 171388 | 35639 | 135748 |  | 12.728868 | 5.275033 | 20.219137 |
| 2003 | 171916 | 35728 | 136188 |  | 12.449385 | 5.156663 | 19.778866 |
| 2004 | 172208 | 35801 | 136407 |  | 12.134339 | 5.028245 | 19.279052 |
| 2005 | 174721 | 36342 | 138379 |  | 11.984012 | 4.970184 | 19.038589 |
| 2006 | 176157 | 36640 | 139517 |  | 11.737215 | 4.868729 | 18.652269 |
| 2007 | 179421 | 37253 | 142168 |  | 11.586994 | 4.793881 | 18.434826 |
| 2008 | 183619 | 37888 | 145731 |  | 11.49026 | 4.717263 | 18.33232 |
| 2009 | 186923 | 38547 | 148376 |  | 11.320862 | 4.638225 | 18.084733 |
| 2010 | 192588 | 39690 | 152898 |  | 11.297766 | 4.620297 | 18.072618 |
| 2011 | 197101 | 40747 | 156354 |  | 11.215203 | 4.596586 | 17.944492 |
| 2012 | 200379 | 41602 | 158777 |  | 11.109793 | 4.570012 | 17.770278 |
| 2013 | 203857 | 42832 | 161026 |  | 11.012042 | 4.581042 | 17.573176 |
| 2014 | 207321 | 44133 | 163188 |  | 10.951163 | 4.612798 | 17.428889 |
| 2015 | 212947 | 46054 | 166892 |  | 10.974086 | 4.69482 | 17.396943 |
| 2016 | 217032 | 47246 | 169785 |  | 10.958691 | 4.719321 | 17.34329 |
| 2017 | 220679 | 48483 | 172196 |  | 10.921369 | 4.748525 | 17.239709 |
| 2018 | 225032 | 49780 | 175252 |  | 10.923086 | 4.78449 | 17.208344 |
| 2019 | 228603 | 50552 | 178051 |  | 10.895613 | 4.772724 | 17.165544 |
| 2020 | 230617 | 50896 | 179721 |  | 10.808376 | 4.725929 | 17.039282 |
| 2021 | 233661 | 51838 | 181823 |  | 10.776062 | 4.734601 | 16.97124 |
| 2022 | 241038 | 54447 | 186591 |  | 10.78806 | 4.829329 | 16.889669 |
| 2023 | 245181 | 55792 | 189390 |  | 10.764476 | 4.854864 | 16.8132 |
| 2024 | 249569 | 57215 | 192354 |  | 10.741162 | 4.880399 | 16.73673 |
| 2025 | 254464 | 58559 | 195905 |  | 10.737483 | 4.897137 | 16.709341 |
| 2026 | 259161 | 59870 | 199291 |  | 10.73355 | 4.913875 | 16.681953 |
| 2027 | 263452 | 61098 | 202354 |  | 10.729174 | 4.930612 | 16.654564 |
| 2028 | 267221 | 62213 | 205007 |  | 10.724513 | 4.94735 | 16.627175 |
| 2029 | 270573 | 63240 | 207334 |  | 10.71973 | 4.964087 | 16.599787 |
| 2030 | 274242 | 64243 | 210000 |  | 10.736307 | 4.982631 | 16.614507 |
| 2031 | 277760 | 65215 | 212545 |  | 10.752538 | 5.001174 | 16.629228 |
| 2032 | 281241 | 66182 | 215059 |  | 10.768317 | 5.019717 | 16.643949 |
| 2033 | 284621 | 67133 | 217487 |  | 10.783329 | 5.038261 | 16.658669 |
| 2034 | 287836 | 68053 | 219782 |  | 10.797693 | 5.056804 | 16.67339 |
| 2035 | 291106 | 68831 | 222275 |  | 10.814283 | 5.065698 | 16.70328 |
| 2036 | 294427 | 69615 | 224812 |  | 10.831148 | 5.074593 | 16.73317 |
| 2037 | 297902 | 70426 | 227476 |  | 10.848688 | 5.083487 | 16.76306 |
| 2038 | 301580 | 71274 | 230306 |  | 10.866758 | 5.092382 | 16.79295 |
| 2039 | 305392 | 72146 | 233246 |  | 10.885068 | 5.101276 | 16.82284 |
| 2040 | 308931 | 73087 | 235844 |  | 10.892376 | 5.114041 | 16.825309 |
| 2041 | 312468 | 74025 | 238443 |  | 10.90021 | 5.126807 | 16.827778 |
| 2042 | 315936 | 74945 | 240991 |  | 10.908682 | 5.139572 | 16.830247 |
| 2043 | 319461 | 75876 | 243585 |  | 10.91782 | 5.152337 | 16.832716 |
| 2044 | 323088 | 76827 | 246261 |  | 10.927585 | 5.165103 | 16.835186 |
| 2045 | 326634 | 77754 | 248880 |  | 10.937978 | 5.177868 | 16.837655 |

Table 2.4. Nordpred projections of case numbers and age-standardized DALYs rate (ASDR) up to the year 2045.

| **DALYs** | Case number | | |  | ASR (per 100,000) | | |
| --- | --- | --- | --- | --- | --- | --- | --- |
| Year | Both | Female | Male |  | Both | Female | Male |
| 1992 | 5951789 | 1229892 | 4721897 |  | 537.009978 | 222.567653 | 848.719044 |
| 1993 | 6066722 | 1249731 | 4816991 |  | 537.527447 | 221.831428 | 850.597135 |
| 1994 | 6133986 | 1262079 | 4871907 |  | 533.61545 | 219.714043 | 845.132909 |
| 1995 | 6175921 | 1272446 | 4903475 |  | 527.735294 | 217.302618 | 836.185288 |
| 1996 | 6173578 | 1281510 | 4892068 |  | 517.320013 | 214.458089 | 818.650724 |
| 1997 | 6190687 | 1290900 | 4899787 |  | 507.888967 | 211.381533 | 803.33816 |
| 1998 | 6217826 | 1302052 | 4915774 |  | 499.474005 | 208.719082 | 789.424391 |
| 1999 | 6253985 | 1310305 | 4943680 |  | 491.377753 | 205.474148 | 776.70531 |
| 2000 | 6281245 | 1312475 | 4968770 |  | 483.093503 | 201.507834 | 764.358715 |
| 2001 | 6289003 | 1312963 | 4976040 |  | 472.728411 | 197.050467 | 748.345677 |
| 2002 | 6310600 | 1318066 | 4992533 |  | 462.604108 | 192.889924 | 732.434794 |
| 2003 | 6334247 | 1321962 | 5012285 |  | 452.391846 | 188.456794 | 716.572031 |
| 2004 | 6350485 | 1325949 | 5024536 |  | 441.169673 | 183.828431 | 698.927045 |
| 2005 | 6447334 | 1346771 | 5100563 |  | 435.737314 | 181.650691 | 690.41627 |
| 2006 | 6501272 | 1357401 | 5143871 |  | 426.956432 | 177.899948 | 676.890312 |
| 2007 | 6618420 | 1379432 | 5238988 |  | 421.804126 | 175.295289 | 669.526345 |
| 2008 | 6761907 | 1402234 | 5359673 |  | 418.161459 | 172.662341 | 665.39521 |
| 2009 | 6870722 | 1425395 | 5445327 |  | 412.049161 | 169.99063 | 656.294032 |
| 2010 | 7061821 | 1465498 | 5596323 |  | 410.864532 | 169.399561 | 655.04815 |
| 2011 | 7210130 | 1500399 | 5709731 |  | 407.639518 | 168.418863 | 650.020548 |
| 2012 | 7319604 | 1529316 | 5790288 |  | 403.623674 | 167.354006 | 643.397668 |
| 2013 | 7442764 | 1574744 | 5868020 |  | 400.298787 | 168.000908 | 636.420073 |
| 2014 | 7561495 | 1622032 | 5939463 |  | 397.975205 | 169.264672 | 630.81611 |
| 2015 | 7745545 | 1688301 | 6057243 |  | 398.30938 | 172.110569 | 628.786801 |
| 2016 | 7891794 | 1731957 | 6159837 |  | 397.971921 | 173.168321 | 627.117099 |
| 2017 | 8020358 | 1776391 | 6243967 |  | 396.77017 | 174.322344 | 623.576551 |
| 2018 | 8166898 | 1820950 | 6345949 |  | 396.576656 | 175.505327 | 622.044564 |
| 2019 | 8292883 | 1847402 | 6445480 |  | 395.647535 | 175.019416 | 620.678771 |
| 2020 | 8374717 | 1862474 | 6512243 |  | 393.11318 | 173.64949 | 617.023926 |
| 2021 | 8489138 | 1896705 | 6592432 |  | 392.404151 | 174.088229 | 615.332181 |
| 2022 | 8754554 | 1989099 | 6765456 |  | 392.676031 | 177.227179 | 612.380658 |
| 2023 | 8899095 | 2035861 | 6863234 |  | 391.990289 | 178.136246 | 609.993418 |
| 2024 | 9049845 | 2084901 | 6964945 |  | 391.310088 | 179.045312 | 607.606178 |
| 2025 | 9218212 | 2131903 | 7086309 |  | 391.325525 | 179.706868 | 606.894335 |
| 2026 | 9380710 | 2178004 | 7202706 |  | 391.330749 | 180.368425 | 606.182493 |
| 2027 | 9531608 | 2221773 | 7309835 |  | 391.320102 | 181.029982 | 605.470651 |
| 2028 | 9667748 | 2262449 | 7405300 |  | 391.295567 | 181.691538 | 604.758808 |
| 2029 | 9791964 | 2300660 | 7491303 |  | 391.262061 | 182.353095 | 604.046966 |
| 2030 | 9926343 | 2338005 | 7588338 |  | 391.881716 | 183.045664 | 604.64963 |
| 2031 | 10055737 | 2374256 | 7681481 |  | 392.493405 | 183.738234 | 605.252295 |
| 2032 | 10183071 | 2410011 | 7773060 |  | 393.09772 | 184.430804 | 605.854959 |
| 2033 | 10306733 | 2444987 | 7861747 |  | 393.6839 | 185.123374 | 606.457624 |
| 2034 | 10425279 | 2478881 | 7946398 |  | 394.253004 | 185.815943 | 607.060288 |
| 2035 | 10543561 | 2507695 | 8035867 |  | 394.833365 | 186.168765 | 608.049789 |
| 2036 | 10662402 | 2536441 | 8125961 |  | 395.427688 | 186.521587 | 609.03929 |
| 2037 | 10784621 | 2565677 | 8218944 |  | 396.049441 | 186.874409 | 610.02879 |
| 2038 | 10911758 | 2595720 | 8316038 |  | 396.695202 | 187.227232 | 611.018291 |
| 2039 | 11042063 | 2626230 | 8415833 |  | 397.355866 | 187.580054 | 612.007792 |
| 2040 | 11159529 | 2655740 | 8503789 |  | 397.569802 | 187.865846 | 612.113977 |
| 2041 | 11275738 | 2684820 | 8590917 |  | 397.805031 | 188.151639 | 612.220162 |
| 2042 | 11388625 | 2713034 | 8675590 |  | 398.063566 | 188.437432 | 612.326347 |
| 2043 | 11501235 | 2741085 | 8760150 |  | 398.348266 | 188.723224 | 612.432532 |
| 2044 | 11615000 | 2769237 | 8845762 |  | 398.659395 | 189.009017 | 612.538717 |
| 2045 | 11725466 | 2796388 | 8929077 |  | 398.994273 | 189.29481 | 612.644902 |

Table 3. The age-standardized rates and AAPCs of Head and Neck Cancers Among Adults Aged 40-64 in 204 Countries and Territories.

|  | Prevalence | |  | Incidence | |  | Mortality | |  | DALYs | |  |
| --- | --- | --- | --- | --- | --- | --- | --- | --- | --- | --- | --- | --- |
| location | ASR in 1990 | ASR in 2021 | AAPC | ASR in 1990 | ASR in 2021 | AAPC | ASR in 1990 | ASR in 2021 | AAPC | ASR in 1990 | ASR in 2021 | AAPC |
| Afghanistan | 36.30 | 33.93 | -0.24 | 10.48 | 8.91 | -0.53 | 9.14 | 7.12 | -0.81 | 326.14 | 257.81 | -0.76 |
| Albania | 55.84 | 73.01 | 0.76 | 14.45 | 15.89 | 0.18 | 10.63 | 8.09 | -1.01 | 377.40 | 294.72 | -0.93 |
| Algeria | 44.59 | 48.34 | 0.27 | 13.09 | 11.97 | -0.29 | 10.36 | 7.40 | -1.09 | 379.40 | 272.07 | -1.09 |
| American Samoa | 25.79 | 24.89 | -0.04 | 7.05 | 7.00 | 0.05 | 5.40 | 5.09 | -0.19 | 198.12 | 188.91 | -0.15 |
| Andorra | 110.10 | 88.16 | -0.78 | 21.72 | 15.63 | -1.15 | 7.57 | 3.98 | -2.21 | 279.20 | 149.05 | -2.16 |
| Angola | 28.69 | 30.47 | 0.15 | 9.83 | 9.64 | -0.11 | 8.44 | 7.48 | -0.37 | 298.47 | 265.46 | -0.37 |
| Antigua and Barbuda | 45.61 | 42.14 | -0.07 | 14.26 | 11.24 | -0.57 | 9.35 | 6.45 | -0.97 | 328.62 | 223.06 | -1.02 |
| Argentina | 84.69 | 51.94 | -1.46 | 21.59 | 11.61 | -1.83 | 13.89 | 5.84 | -2.63 | 495.18 | 204.44 | -2.68 |
| Armenia | 68.75 | 45.43 | -1.35 | 15.91 | 10.25 | -1.44 | 11.43 | 6.02 | -2.10 | 402.88 | 213.00 | -2.10 |
| Australia | 158.88 | 165.37 | 0.09 | 29.85 | 26.77 | -0.25 | 8.12 | 4.36 | -1.93 | 292.19 | 164.94 | -1.76 |
| Austria | 114.60 | 107.47 | -0.24 | 27.69 | 22.33 | -0.67 | 13.32 | 7.19 | -2.02 | 490.73 | 256.74 | -2.12 |
| Azerbaijan | 46.80 | 33.34 | -1.11 | 11.58 | 7.71 | -1.25 | 9.08 | 5.13 | -1.79 | 323.18 | 181.31 | -1.82 |
| Bahamas | 77.10 | 72.33 | -0.09 | 25.54 | 20.52 | -0.45 | 17.52 | 12.69 | -0.89 | 626.81 | 457.84 | -0.87 |
| Bahrain | 46.32 | 41.42 | -0.29 | 11.50 | 8.22 | -1.05 | 7.79 | 3.60 | -2.44 | 271.65 | 129.37 | -2.32 |
| Bangladesh | 93.26 | 87.52 | -0.15 | 37.45 | 29.38 | -0.75 | 31.10 | 19.60 | -1.44 | 1103.05 | 698.43 | -1.43 |
| Barbados | 48.33 | 50.57 | 0.24 | 15.71 | 13.58 | -0.37 | 10.05 | 7.65 | -0.79 | 357.35 | 269.41 | -0.82 |
| Belarus | 118.54 | 158.06 | 0.95 | 33.83 | 46.30 | 1.05 | 22.11 | 22.01 | 0.04 | 806.17 | 797.28 | 0.01 |
| Belgium | 175.29 | 149.16 | -0.65 | 34.14 | 27.29 | -0.84 | 12.97 | 7.48 | -1.91 | 481.79 | 273.40 | -1.96 |
| Belize | 26.55 | 32.84 | 0.62 | 8.06 | 8.86 | 0.21 | 5.77 | 5.80 | -0.08 | 200.92 | 205.10 | -0.03 |
| Benin | 14.75 | 17.91 | 0.65 | 4.65 | 5.31 | 0.41 | 3.79 | 4.04 | 0.19 | 133.90 | 143.06 | 0.20 |
| Bermuda | 96.29 | 98.80 | 0.09 | 27.50 | 20.58 | -1.02 | 15.38 | 7.47 | -2.45 | 543.14 | 268.71 | -2.38 |
| Bhutan | 70.51 | 74.37 | 0.20 | 28.69 | 25.75 | -0.33 | 23.57 | 17.50 | -0.94 | 846.59 | 628.97 | -0.95 |
| Bolivia (Plurinational State of) | 20.02 | 18.85 | -0.18 | 6.72 | 5.50 | -0.64 | 5.52 | 3.79 | -1.20 | 195.01 | 134.71 | -1.18 |
| Bosnia and Herzegovina | 83.64 | 98.34 | 0.64 | 21.18 | 23.17 | 0.29 | 14.54 | 11.84 | -0.67 | 521.74 | 421.42 | -0.70 |
| Botswana | 54.08 | 46.53 | -0.48 | 18.07 | 15.28 | -0.53 | 14.17 | 11.05 | -0.78 | 506.62 | 395.47 | -0.78 |
| Brazil | 64.01 | 73.17 | 0.46 | 20.44 | 20.33 | 0.00 | 14.73 | 12.07 | -0.62 | 531.15 | 431.75 | -0.65 |
| Brunei Darussalam | 67.65 | 62.49 | -0.25 | 21.79 | 19.03 | -0.43 | 14.91 | 11.13 | -0.93 | 530.72 | 405.27 | -0.86 |
| Bulgaria | 110.16 | 187.07 | 1.85 | 25.29 | 40.20 | 1.58 | 13.65 | 16.69 | 0.75 | 505.53 | 605.22 | 0.71 |
| Burkina Faso | 15.61 | 19.73 | 0.78 | 4.90 | 5.91 | 0.62 | 3.99 | 4.63 | 0.51 | 141.25 | 164.23 | 0.51 |
| Burundi | 54.08 | 38.13 | -1.13 | 20.76 | 13.69 | -1.34 | 17.92 | 11.45 | -1.47 | 644.62 | 409.27 | -1.46 |
| Cabo Verde | 15.95 | 61.39 | 4.49 | 3.58 | 16.24 | 5.09 | 2.76 | 9.49 | 4.17 | 94.80 | 346.67 | 4.29 |
| Cambodia | 41.74 | 50.17 | 0.64 | 14.01 | 14.55 | 0.15 | 11.85 | 10.63 | -0.32 | 424.08 | 382.03 | -0.31 |
| Cameroon | 17.80 | 23.85 | 0.94 | 5.53 | 7.00 | 0.75 | 4.41 | 5.24 | 0.53 | 156.82 | 186.37 | 0.55 |
| Canada | 151.92 | 103.36 | -1.06 | 28.25 | 17.93 | -1.33 | 8.51 | 4.03 | -2.44 | 306.50 | 148.36 | -2.35 |
| Central African Republic | 31.34 | 27.25 | -0.45 | 11.16 | 9.54 | -0.52 | 9.73 | 8.24 | -0.54 | 344.81 | 292.82 | -0.53 |
| Chad | 12.02 | 19.89 | 1.68 | 3.83 | 6.17 | 1.59 | 3.17 | 5.03 | 1.55 | 111.91 | 177.04 | 1.54 |
| Chile | 38.83 | 35.22 | -0.31 | 10.48 | 7.14 | -1.24 | 6.40 | 2.69 | -2.80 | 227.10 | 97.63 | -2.73 |
| China | 60.49 | 82.68 | 0.99 | 17.84 | 15.94 | -0.36 | 14.03 | 6.32 | -2.54 | 515.86 | 234.60 | -2.52 |
| Colombia | 34.51 | 25.00 | -1.10 | 9.22 | 5.72 | -1.57 | 6.53 | 2.84 | -2.62 | 228.49 | 100.72 | -2.58 |
| Comoros | 46.61 | 40.70 | -0.53 | 16.82 | 13.87 | -0.70 | 13.98 | 10.87 | -0.79 | 500.68 | 389.40 | -0.79 |
| Congo | 34.19 | 33.69 | -0.06 | 11.67 | 10.43 | -0.31 | 9.71 | 7.83 | -0.63 | 347.06 | 278.99 | -0.64 |
| Cook Islands | 18.85 | 29.26 | 1.38 | 4.81 | 6.27 | 0.84 | 2.86 | 2.69 | -0.20 | 105.61 | 101.67 | -0.13 |
| Costa Rica | 42.42 | 31.11 | -1.26 | 11.03 | 7.50 | -1.52 | 6.59 | 3.66 | -2.03 | 233.46 | 130.91 | -2.04 |
| Coted'Ivoire | 25.23 | 28.21 | 0.35 | 8.25 | 8.62 | 0.14 | 6.64 | 6.40 | -0.12 | 236.84 | 230.24 | -0.09 |
| Croatia | 179.69 | 158.13 | -0.34 | 45.28 | 34.56 | -0.88 | 25.47 | 13.63 | -1.99 | 926.81 | 490.30 | -2.05 |
| Cuba | 96.50 | 150.18 | 1.60 | 23.60 | 31.97 | 1.15 | 13.52 | 15.21 | 0.47 | 478.11 | 534.71 | 0.66 |
| Cyprus | 51.78 | 69.57 | 1.05 | 10.15 | 11.54 | 0.51 | 4.81 | 3.13 | -1.31 | 171.31 | 115.36 | -1.21 |
| Czechia | 91.51 | 107.64 | 0.51 | 25.49 | 26.22 | 0.17 | 16.59 | 11.53 | -1.19 | 611.07 | 406.58 | -1.25 |
| Democratic People's Republic of Korea | 41.89 | 46.81 | 0.37 | 11.94 | 11.76 | -0.05 | 8.89 | 7.45 | -0.56 | 326.97 | 273.33 | -0.57 |
| Democratic Republic of the Congo | 23.52 | 25.58 | 0.26 | 7.91 | 8.07 | 0.03 | 6.61 | 6.37 | -0.15 | 233.89 | 225.52 | -0.14 |
| Denmark | 112.26 | 124.02 | 0.32 | 23.00 | 23.59 | 0.11 | 9.17 | 6.43 | -1.06 | 335.13 | 231.88 | -1.08 |
| Djibouti | 51.15 | 48.61 | -0.16 | 18.16 | 16.36 | -0.40 | 14.90 | 12.74 | -0.54 | 534.85 | 454.88 | -0.56 |
| Dominica | 47.54 | 55.37 | 0.48 | 16.60 | 17.39 | 0.14 | 11.88 | 11.90 | 0.01 | 414.63 | 418.49 | 0.02 |
| Dominican Republic | 33.95 | 44.61 | 0.98 | 11.73 | 14.10 | 0.70 | 8.85 | 9.37 | 0.31 | 314.76 | 334.89 | 0.34 |
| Ecuador | 13.96 | 15.39 | 0.42 | 4.38 | 4.02 | -0.32 | 3.27 | 2.28 | -1.25 | 116.91 | 81.56 | -1.23 |
| Egypt | 14.67 | 21.69 | 1.29 | 3.62 | 4.83 | 0.99 | 2.79 | 2.75 | -0.01 | 99.40 | 97.72 | 0.00 |
| El Salvador | 17.18 | 24.03 | 1.24 | 5.62 | 6.23 | 0.35 | 4.19 | 3.48 | -0.52 | 149.00 | 125.21 | -0.49 |
| Equatorial Guinea | 29.47 | 33.87 | 0.52 | 10.21 | 9.73 | -0.13 | 8.80 | 6.54 | -0.93 | 311.79 | 234.13 | -0.89 |
| Eritrea | 51.68 | 45.62 | -0.38 | 20.09 | 16.44 | -0.67 | 17.33 | 13.54 | -0.82 | 628.05 | 490.28 | -0.83 |
| Estonia | 115.79 | 136.91 | 0.81 | 30.01 | 30.54 | 0.34 | 16.76 | 10.98 | -1.13 | 609.49 | 390.60 | -1.17 |
| Eswatini | 58.19 | 63.46 | 0.30 | 19.02 | 21.21 | 0.36 | 15.04 | 16.15 | 0.23 | 533.47 | 581.21 | 0.28 |
| Ethiopia | 35.80 | 28.25 | -0.78 | 13.71 | 9.42 | -1.21 | 12.26 | 7.54 | -1.57 | 436.15 | 268.01 | -1.57 |
| Fiji | 26.29 | 25.75 | -0.11 | 7.67 | 7.27 | -0.20 | 5.17 | 4.69 | -0.33 | 191.02 | 173.61 | -0.32 |
| Finland | 74.12 | 85.44 | 0.54 | 14.94 | 15.15 | 0.16 | 5.71 | 3.77 | -1.27 | 211.07 | 139.08 | -1.27 |
| France | 346.02 | 269.29 | -0.71 | 75.78 | 46.93 | -1.46 | 29.58 | 9.85 | -3.38 | 1095.04 | 363.52 | -3.39 |
| Gabon | 36.26 | 38.73 | 0.24 | 12.02 | 11.46 | -0.13 | 9.62 | 8.08 | -0.56 | 342.64 | 287.33 | -0.54 |
| Gambia | 13.32 | 16.32 | 0.75 | 4.58 | 5.36 | 0.44 | 3.54 | 3.84 | 0.26 | 126.96 | 138.41 | 0.28 |
| Georgia | 84.93 | 76.63 | -0.39 | 20.92 | 19.78 | -0.29 | 14.40 | 12.79 | -0.44 | 516.41 | 451.87 | -0.48 |
| Germany | 139.41 | 111.17 | -0.72 | 32.59 | 23.81 | -1.03 | 14.82 | 7.66 | -2.09 | 558.50 | 271.49 | -2.32 |
| Ghana | 10.91 | 13.94 | 0.79 | 2.93 | 3.30 | 0.38 | 2.37 | 2.45 | 0.12 | 84.27 | 84.68 | 0.03 |
| Greece | 111.71 | 122.80 | 0.21 | 19.22 | 20.81 | 0.19 | 6.81 | 6.25 | -0.30 | 249.16 | 229.49 | -0.30 |
| Greenland | 191.14 | 123.10 | -1.31 | 65.95 | 36.00 | -1.87 | 47.76 | 20.97 | -2.51 | 1734.86 | 751.38 | -2.67 |
| Grenada | 47.69 | 43.36 | -0.19 | 16.88 | 12.90 | -0.75 | 12.21 | 8.26 | -1.12 | 434.25 | 291.29 | -1.16 |
| Guam | 40.39 | 52.78 | 0.90 | 10.62 | 13.07 | 0.66 | 7.38 | 7.51 | 0.08 | 271.30 | 288.67 | 0.21 |
| Guatemala | 17.90 | 12.98 | -1.11 | 6.08 | 4.02 | -1.45 | 4.94 | 2.70 | -2.05 | 175.42 | 96.77 | -2.01 |
| Guinea | 27.14 | 36.34 | 0.96 | 10.31 | 12.79 | 0.70 | 8.17 | 9.75 | 0.57 | 291.61 | 348.20 | 0.57 |
| Guinea-Bissau | 19.67 | 25.26 | 0.82 | 6.58 | 7.99 | 0.64 | 5.62 | 6.48 | 0.47 | 198.97 | 229.87 | 0.48 |
| Guyana | 23.98 | 26.87 | 0.50 | 9.02 | 8.75 | 0.10 | 7.19 | 6.45 | -0.07 | 252.16 | 230.88 | 0.02 |
| Haiti | 39.86 | 34.41 | -0.45 | 15.01 | 12.13 | -0.62 | 13.16 | 10.17 | -0.81 | 462.71 | 358.68 | -0.79 |
| Honduras | 15.59 | 21.15 | 1.03 | 4.96 | 6.18 | 0.76 | 3.94 | 4.41 | 0.41 | 139.80 | 154.40 | 0.36 |
| Hungary | 183.07 | 226.32 | 0.65 | 52.74 | 55.70 | 0.13 | 33.19 | 25.73 | -0.95 | 1244.20 | 905.06 | -1.06 |
| Iceland | 94.68 | 97.28 | 0.10 | 17.32 | 16.26 | -0.19 | 5.64 | 3.64 | -1.43 | 207.39 | 138.23 | -1.33 |
| India | 82.00 | 102.87 | 0.74 | 31.59 | 35.49 | 0.37 | 25.40 | 23.87 | -0.19 | 913.95 | 861.80 | -0.19 |
| Indonesia | 31.85 | 35.46 | 0.36 | 10.11 | 10.00 | -0.03 | 8.00 | 6.95 | -0.45 | 288.96 | 249.57 | -0.47 |
| Iran (Islamic Republic of) | 34.35 | 41.19 | 0.50 | 7.41 | 7.38 | -0.10 | 5.14 | 3.29 | -1.42 | 182.09 | 118.58 | -1.39 |
| Iraq | 44.44 | 49.86 | 0.37 | 11.19 | 10.61 | -0.16 | 8.01 | 5.52 | -1.19 | 287.12 | 198.25 | -1.18 |
| Ireland | 116.92 | 113.30 | -0.23 | 23.58 | 19.56 | -0.68 | 9.53 | 4.70 | -2.20 | 343.59 | 175.66 | -2.07 |
| Israel | 53.24 | 57.75 | 0.21 | 10.44 | 10.04 | -0.19 | 4.84 | 3.10 | -1.52 | 175.14 | 113.59 | -1.48 |
| Italy | 146.07 | 100.51 | -1.24 | 31.13 | 18.75 | -1.69 | 13.23 | 5.52 | -2.82 | 477.75 | 200.07 | -2.83 |
| Jamaica | 30.24 | 33.29 | 0.41 | 8.88 | 8.82 | 0.06 | 5.77 | 5.34 | -0.16 | 201.19 | 188.28 | -0.13 |
| Japan | 54.08 | 66.49 | 0.60 | 11.49 | 13.62 | 0.49 | 3.42 | 3.15 | -0.29 | 124.26 | 114.74 | -0.28 |
| Jordan | 36.57 | 32.69 | -0.33 | 9.49 | 7.00 | -0.94 | 6.57 | 3.34 | -2.15 | 236.35 | 122.09 | -2.10 |
| Kazakhstan | 77.84 | 50.95 | -1.32 | 23.27 | 13.36 | -1.78 | 17.20 | 8.10 | -2.38 | 612.83 | 288.53 | -2.39 |
| Kenya | 42.78 | 58.19 | 0.97 | 14.90 | 19.02 | 0.78 | 11.80 | 14.33 | 0.61 | 425.37 | 517.72 | 0.62 |
| Kiribati | 33.67 | 39.26 | 0.51 | 13.94 | 15.30 | 0.31 | 11.23 | 11.79 | 0.18 | 419.99 | 441.95 | 0.18 |
| Kuwait | 69.10 | 22.48 | -3.66 | 15.70 | 4.12 | -3.84 | 7.87 | 1.45 | -5.02 | 281.94 | 53.30 | -4.98 |
| Kyrgyzstan | 64.85 | 32.37 | -2.33 | 20.09 | 9.34 | -2.64 | 15.06 | 6.16 | -3.09 | 547.92 | 220.02 | -3.16 |
| Lao People's Democratic Republic | 40.47 | 34.12 | -0.53 | 14.38 | 10.67 | -0.95 | 12.51 | 8.19 | -1.34 | 449.67 | 294.46 | -1.34 |
| Latvia | 93.31 | 102.67 | 0.33 | 27.29 | 29.36 | 0.28 | 18.95 | 15.78 | -0.54 | 690.15 | 563.86 | -0.64 |
| Lebanon | 56.71 | 62.75 | 0.42 | 13.16 | 11.64 | -0.29 | 8.46 | 4.65 | -1.85 | 301.91 | 169.47 | -1.78 |
| Lesotho | 40.03 | 63.46 | 1.51 | 13.26 | 22.48 | 1.72 | 10.62 | 18.14 | 1.77 | 375.08 | 649.42 | 1.81 |
| Liberia | 13.77 | 19.95 | 1.17 | 4.42 | 5.74 | 0.85 | 3.64 | 4.23 | 0.47 | 128.74 | 150.79 | 0.50 |
| Libya | 75.28 | 96.82 | 0.82 | 18.94 | 21.30 | 0.33 | 13.81 | 12.81 | -0.22 | 500.51 | 467.46 | -0.20 |
| Lithuania | 98.27 | 108.03 | 0.28 | 28.75 | 34.73 | 0.62 | 18.80 | 18.84 | 0.01 | 688.99 | 673.77 | -0.08 |
| Luxembourg | 179.13 | 143.88 | -0.87 | 38.27 | 26.34 | -1.34 | 16.22 | 7.00 | -2.78 | 595.43 | 256.94 | -2.78 |
| Madagascar | 42.63 | 32.86 | -0.85 | 15.62 | 11.50 | -1.00 | 13.01 | 9.22 | -1.11 | 466.38 | 330.80 | -1.11 |
| Malawi | 19.10 | 24.03 | 0.78 | 7.07 | 8.14 | 0.48 | 5.63 | 6.04 | 0.25 | 202.00 | 217.98 | 0.27 |
| Malaysia | 99.29 | 114.77 | 0.50 | 30.31 | 28.83 | -0.14 | 23.68 | 17.93 | -0.88 | 866.36 | 664.15 | -0.84 |
| Maldives | 31.11 | 30.46 | -0.31 | 9.79 | 6.96 | -1.21 | 7.03 | 3.23 | -2.60 | 247.85 | 116.84 | -2.51 |
| Mali | 19.32 | 20.30 | 0.18 | 6.55 | 6.47 | -0.02 | 5.31 | 4.92 | -0.22 | 189.86 | 176.63 | -0.21 |
| Malta | 105.97 | 119.95 | 0.36 | 21.46 | 21.43 | -0.01 | 9.95 | 6.41 | -1.45 | 354.91 | 239.93 | -1.30 |
| Marshall Islands | 24.58 | 27.63 | 0.38 | 8.16 | 8.71 | 0.20 | 6.60 | 6.55 | -0.03 | 243.67 | 245.24 | 0.01 |
| Mauritania | 16.03 | 21.89 | 1.03 | 5.04 | 5.91 | 0.52 | 4.04 | 3.95 | -0.06 | 141.86 | 139.56 | -0.05 |
| Mauritius | 64.34 | 68.20 | 0.30 | 16.12 | 16.18 | 0.26 | 10.07 | 8.69 | -0.32 | 358.45 | 314.26 | -0.27 |
| Mexico | 22.30 | 18.56 | -0.78 | 6.05 | 4.66 | -1.02 | 4.40 | 2.69 | -1.79 | 153.93 | 95.27 | -1.75 |
| Micronesia (Federated States of) | 26.96 | 29.36 | 0.27 | 9.12 | 9.03 | -0.04 | 7.40 | 6.54 | -0.40 | 274.72 | 243.81 | -0.39 |
| Monaco | 249.06 | 220.08 | -0.40 | 41.19 | 34.82 | -0.54 | 13.04 | 8.60 | -1.35 | 481.18 | 325.94 | -1.26 |
| Mongolia | 34.66 | 28.26 | -0.70 | 12.91 | 8.97 | -1.21 | 10.74 | 6.45 | -1.68 | 380.03 | 232.21 | -1.60 |
| Montenegro | 172.58 | 184.08 | 0.21 | 35.48 | 35.70 | 0.03 | 18.31 | 15.56 | -0.45 | 666.42 | 564.03 | -0.47 |
| Morocco | 35.87 | 42.70 | 0.57 | 9.97 | 10.39 | 0.16 | 8.12 | 6.99 | -0.49 | 292.97 | 249.63 | -0.49 |
| Mozambique | 25.50 | 29.85 | 0.52 | 8.50 | 10.16 | 0.59 | 7.21 | 8.25 | 0.45 | 253.38 | 293.06 | 0.49 |
| Myanmar | 37.86 | 31.80 | -0.56 | 12.90 | 9.38 | -1.03 | 10.86 | 6.78 | -1.52 | 389.53 | 243.28 | -1.52 |
| Namibia | 67.80 | 91.20 | 0.98 | 24.22 | 29.33 | 0.69 | 18.70 | 20.14 | 0.29 | 670.88 | 731.00 | 0.32 |
| Nauru | 32.86 | 34.97 | 0.20 | 10.74 | 10.63 | -0.04 | 8.39 | 7.58 | -0.33 | 311.19 | 283.66 | -0.30 |
| Nepal | 68.40 | 76.35 | 0.38 | 27.24 | 26.82 | -0.04 | 22.59 | 18.95 | -0.55 | 807.74 | 680.04 | -0.54 |
| Netherlands | 132.37 | 126.49 | -0.16 | 23.39 | 20.56 | -0.43 | 7.11 | 4.09 | -1.77 | 262.12 | 152.14 | -1.74 |
| New Zealand | 102.80 | 92.86 | -0.39 | 19.09 | 15.70 | -0.69 | 5.83 | 3.30 | -1.80 | 208.45 | 123.09 | -1.68 |
| Nicaragua | 15.40 | 16.02 | 0.01 | 4.36 | 4.09 | -0.27 | 3.27 | 2.53 | -0.91 | 115.03 | 88.86 | -0.92 |
| Niger | 12.61 | 14.48 | 0.49 | 4.00 | 4.43 | 0.37 | 3.35 | 3.52 | 0.19 | 118.34 | 123.78 | 0.18 |
| Nigeria | 24.81 | 22.04 | -0.42 | 7.68 | 6.66 | -0.49 | 6.76 | 5.38 | -0.75 | 243.77 | 195.70 | -0.72 |
| Niue | 26.31 | 31.21 | 0.53 | 7.70 | 8.20 | 0.19 | 5.45 | 5.02 | -0.26 | 201.46 | 185.96 | -0.27 |
| North Macedonia | 83.24 | 101.58 | 0.60 | 19.98 | 21.66 | 0.26 | 14.02 | 11.49 | -0.71 | 505.58 | 409.50 | -0.76 |
| Northern Mariana Islands | 67.92 | 121.99 | 1.85 | 18.52 | 29.59 | 1.52 | 11.16 | 15.06 | 1.00 | 417.77 | 568.65 | 1.03 |
| Norway | 74.94 | 70.71 | -0.10 | 14.72 | 12.05 | -0.57 | 5.07 | 2.61 | -2.15 | 182.00 | 95.28 | -2.10 |
| Oman | 27.29 | 29.68 | 0.21 | 7.80 | 6.57 | -0.69 | 5.26 | 2.98 | -1.91 | 186.41 | 106.68 | -1.84 |
| Pakistan | 132.87 | 161.91 | 0.65 | 51.02 | 59.24 | 0.49 | 39.68 | 42.34 | 0.21 | 1422.51 | 1530.25 | 0.25 |
| Palau | 169.55 | 176.09 | 0.11 | 46.00 | 43.81 | -0.20 | 25.26 | 22.28 | -0.44 | 966.29 | 862.16 | -0.38 |
| Palestine | 19.75 | 21.34 | 0.23 | 4.66 | 4.31 | -0.26 | 3.27 | 2.28 | -1.16 | 115.24 | 80.58 | -1.16 |
| Panama | 27.65 | 25.54 | -0.36 | 8.10 | 6.62 | -0.80 | 5.39 | 3.49 | -1.44 | 187.38 | 122.89 | -1.39 |
| Papua New Guinea | 18.08 | 18.51 | 0.09 | 6.00 | 5.97 | 0.05 | 4.79 | 4.56 | -0.09 | 176.99 | 168.95 | -0.08 |
| Paraguay | 29.90 | 48.72 | 1.59 | 9.14 | 13.80 | 1.28 | 6.45 | 8.46 | 0.98 | 229.64 | 301.40 | 0.98 |
| Peru | 15.40 | 18.32 | 0.67 | 4.78 | 4.34 | -0.05 | 3.53 | 2.15 | -1.51 | 125.49 | 77.38 | -1.47 |
| Philippines | 35.64 | 35.87 | 0.03 | 11.29 | 10.85 | -0.15 | 8.32 | 7.67 | -0.26 | 303.46 | 278.09 | -0.29 |
| Poland | 95.18 | 111.44 | 0.47 | 27.08 | 30.39 | 0.27 | 19.89 | 16.23 | -0.79 | 726.90 | 573.10 | -0.90 |
| Portugal | 111.05 | 193.72 | 1.85 | 27.30 | 38.59 | 1.11 | 15.94 | 12.19 | -0.80 | 581.36 | 446.36 | -0.79 |
| Puerto Rico | 74.80 | 56.16 | -1.07 | 20.61 | 12.32 | -1.81 | 11.73 | 5.19 | -2.84 | 418.45 | 186.79 | -2.81 |
| Qatar | 39.73 | 44.86 | 0.40 | 9.21 | 7.93 | -0.41 | 5.81 | 2.77 | -2.41 | 200.48 | 99.78 | -2.26 |
| Republic of Korea | 59.44 | 58.42 | -0.10 | 13.19 | 11.02 | -0.63 | 6.54 | 2.52 | -3.04 | 233.36 | 93.53 | -2.92 |
| Republic of Moldova | 92.61 | 108.13 | 0.60 | 30.09 | 35.11 | 0.53 | 21.89 | 20.75 | -0.20 | 794.44 | 733.65 | -0.29 |
| Romania | 91.71 | 195.22 | 2.54 | 25.19 | 50.17 | 2.34 | 17.55 | 25.52 | 1.25 | 649.72 | 915.14 | 1.12 |
| Russian Federation | 107.09 | 125.04 | 0.79 | 33.36 | 35.55 | 0.51 | 19.57 | 13.87 | -0.83 | 714.63 | 506.26 | -0.85 |
| Rwanda | 60.62 | 45.11 | -1.28 | 23.36 | 15.51 | -1.33 | 20.31 | 12.14 | -1.66 | 730.43 | 434.04 | -1.68 |
| Saint Kitts and Nevis | 39.94 | 43.60 | 0.25 | 14.53 | 12.40 | -0.39 | 11.10 | 7.98 | -0.97 | 396.18 | 279.01 | -1.04 |
| Saint Lucia | 60.86 | 61.50 | 0.10 | 19.95 | 16.58 | -0.52 | 14.31 | 10.25 | -0.99 | 506.33 | 368.03 | -0.93 |
| Saint Vincent and the Grenadines | 62.18 | 74.63 | 0.45 | 20.56 | 21.78 | 0.06 | 14.47 | 14.16 | -0.19 | 514.73 | 511.31 | -0.11 |
| Samoa | 28.04 | 31.46 | 0.37 | 9.16 | 9.28 | 0.04 | 7.13 | 6.57 | -0.26 | 262.76 | 244.80 | -0.23 |
| San Marino | 164.02 | 96.16 | -1.86 | 29.22 | 16.09 | -2.07 | 9.39 | 4.21 | -2.77 | 345.73 | 157.27 | -2.73 |
| Sao Tome and Principe | 6.75 | 8.99 | 0.92 | 1.54 | 1.93 | 0.71 | 1.28 | 1.40 | 0.29 | 42.82 | 47.05 | 0.30 |
| Saudi Arabia | 32.34 | 42.56 | 0.89 | 9.82 | 9.84 | 0.04 | 7.55 | 4.97 | -1.37 | 273.94 | 183.77 | -1.31 |
| Senegal | 16.31 | 21.93 | 1.03 | 4.94 | 6.24 | 0.81 | 3.97 | 4.61 | 0.61 | 140.69 | 163.37 | 0.60 |
| Serbia | 109.32 | 142.21 | 0.97 | 28.58 | 31.33 | 0.43 | 18.48 | 14.07 | -0.77 | 665.21 | 505.10 | -0.78 |
| Seychelles | 136.17 | 158.76 | 0.56 | 40.13 | 42.28 | 0.25 | 27.89 | 24.37 | -0.35 | 1001.84 | 889.85 | -0.33 |
| Sierra Leone | 14.82 | 19.64 | 0.92 | 4.60 | 5.80 | 0.75 | 3.77 | 4.46 | 0.54 | 133.39 | 158.09 | 0.56 |
| Singapore | 111.66 | 70.88 | -1.32 | 29.40 | 14.70 | -2.08 | 18.96 | 5.52 | -3.81 | 699.59 | 199.38 | -3.88 |
| Slovakia | 183.08 | 161.80 | -0.33 | 54.72 | 44.52 | -0.57 | 33.96 | 22.23 | -1.38 | 1261.86 | 798.40 | -1.50 |
| Slovenia | 146.63 | 138.66 | -0.32 | 38.14 | 30.77 | -0.95 | 20.76 | 10.88 | -2.45 | 752.08 | 392.51 | -2.23 |
| Solomon Islands | 22.17 | 26.66 | 0.58 | 7.44 | 8.37 | 0.36 | 6.17 | 6.44 | 0.11 | 227.95 | 241.16 | 0.16 |
| Somalia | 46.70 | 38.13 | -0.63 | 17.49 | 13.95 | -0.71 | 15.22 | 12.13 | -0.72 | 547.20 | 434.15 | -0.73 |
| South Africa | 57.94 | 55.52 | -0.11 | 17.72 | 16.82 | -0.26 | 12.64 | 11.11 | -0.51 | 462.70 | 397.81 | -0.59 |
| South Sudan | 44.98 | 39.53 | -0.39 | 16.48 | 13.95 | -0.52 | 13.89 | 11.34 | -0.63 | 493.28 | 404.09 | -0.62 |
| Spain | 267.00 | 192.01 | -1.04 | 49.68 | 32.54 | -1.38 | 18.31 | 7.72 | -2.89 | 680.46 | 279.73 | -2.89 |
| Sri Lanka | 52.02 | 95.51 | 2.02 | 16.63 | 22.49 | 1.07 | 11.05 | 10.50 | -0.10 | 395.77 | 380.23 | -0.07 |
| Sudan | 28.75 | 27.45 | -0.17 | 7.69 | 6.29 | -0.66 | 6.33 | 4.22 | -1.30 | 226.44 | 151.39 | -1.30 |
| Suriname | 22.88 | 24.95 | 0.34 | 8.03 | 7.81 | -0.03 | 6.15 | 5.65 | -0.22 | 218.54 | 201.40 | -0.21 |
| Sweden | 60.49 | 60.13 | -0.10 | 11.89 | 10.66 | -0.43 | 4.00 | 2.59 | -1.45 | 143.49 | 93.65 | -1.41 |
| Switzerland | 116.18 | 91.24 | -0.93 | 22.83 | 17.13 | -1.07 | 8.82 | 4.68 | -2.08 | 323.26 | 169.84 | -2.14 |
| Syrian Arab Republic | 22.12 | 27.59 | 0.71 | 5.28 | 5.47 | 0.07 | 3.75 | 2.65 | -1.16 | 134.23 | 94.84 | -1.16 |
| Taiwan (Province of China) | 168.68 | 365.67 | 2.58 | 39.77 | 74.41 | 2.05 | 21.65 | 25.11 | 0.58 | 817.03 | 954.24 | 0.51 |
| Tajikistan | 29.62 | 17.36 | -1.74 | 8.76 | 5.09 | -1.78 | 7.15 | 3.92 | -1.96 | 255.57 | 140.98 | -1.93 |
| Thailand | 76.36 | 99.33 | 0.91 | 20.82 | 21.66 | 0.19 | 13.52 | 10.03 | -0.89 | 492.28 | 375.11 | -0.82 |
| Timor-Leste | 26.88 | 29.63 | 0.27 | 9.06 | 9.08 | -0.02 | 7.57 | 6.87 | -0.35 | 270.54 | 245.72 | -0.36 |
| Togo | 16.86 | 24.58 | 1.21 | 5.13 | 7.12 | 1.07 | 4.08 | 5.35 | 0.91 | 144.68 | 189.56 | 0.91 |
| Tokelau | 24.27 | 29.37 | 0.61 | 7.59 | 7.81 | 0.08 | 5.76 | 4.88 | -0.55 | 210.36 | 181.80 | -0.47 |
| Tonga | 21.60 | 26.13 | 0.59 | 6.28 | 7.06 | 0.35 | 4.44 | 4.49 | 0.05 | 162.94 | 166.23 | 0.08 |
| Trinidad and Tobago | 33.70 | 36.59 | 0.46 | 11.30 | 10.29 | -0.12 | 8.06 | 6.39 | -0.66 | 283.90 | 227.23 | -0.64 |
| Tunisia | 65.45 | 84.89 | 0.83 | 16.95 | 18.08 | 0.20 | 12.15 | 9.39 | -0.84 | 438.78 | 343.10 | -0.80 |
| Turkey | 53.10 | 51.06 | -0.14 | 12.94 | 9.71 | -0.94 | 9.66 | 4.47 | -2.48 | 344.56 | 159.53 | -2.47 |
| Turkmenistan | 55.52 | 42.34 | -0.82 | 18.09 | 13.49 | -0.85 | 14.11 | 9.35 | -1.21 | 503.53 | 335.20 | -1.20 |
| Tuvalu | 22.98 | 28.04 | 0.64 | 7.66 | 8.16 | 0.21 | 6.21 | 5.74 | -0.25 | 228.72 | 214.40 | -0.20 |
| Uganda | 61.99 | 64.54 | 0.08 | 24.15 | 23.96 | -0.07 | 20.46 | 19.04 | -0.26 | 737.35 | 690.55 | -0.24 |
| Ukraine | 118.54 | 119.71 | 0.02 | 31.74 | 35.13 | 0.46 | 18.93 | 17.85 | -0.15 | 692.74 | 648.71 | -0.20 |
| United Arab Emirates | 41.44 | 27.12 | -1.66 | 12.16 | 6.56 | -2.18 | 8.65 | 3.59 | -2.74 | 303.23 | 126.59 | -2.72 |
| United Kingdom | 99.00 | 146.55 | 1.21 | 18.78 | 25.79 | 0.93 | 6.22 | 5.82 | -0.32 | 224.45 | 216.29 | -0.23 |
| United Republic of Tanzania | 50.88 | 41.75 | -0.65 | 18.24 | 14.26 | -0.81 | 14.96 | 11.07 | -0.99 | 536.19 | 396.74 | -0.98 |
| United States of America | 180.20 | 145.86 | -0.71 | 31.51 | 24.05 | -0.90 | 7.99 | 4.62 | -1.79 | 292.30 | 170.49 | -1.77 |
| United States Virgin Islands | 47.41 | 33.96 | -1.24 | 14.35 | 9.24 | -1.55 | 9.40 | 5.26 | -1.94 | 335.04 | 188.51 | -1.92 |
| Uruguay | 134.03 | 103.30 | -0.89 | 33.45 | 22.36 | -1.32 | 19.81 | 10.24 | -2.19 | 696.32 | 362.51 | -2.18 |
| Uzbekistan | 44.10 | 26.96 | -1.74 | 12.30 | 8.32 | -1.37 | 9.40 | 5.75 | -1.61 | 337.33 | 205.38 | -1.64 |
| Vanuatu | 19.58 | 20.57 | 0.14 | 6.54 | 6.69 | 0.06 | 5.24 | 5.10 | -0.09 | 193.71 | 190.46 | -0.06 |
| Venezuela (Bolivarian Republic of) | 38.39 | 39.97 | 0.08 | 10.53 | 9.89 | -0.47 | 7.56 | 5.94 | -0.92 | 263.45 | 208.41 | -0.90 |
| Viet Nam | 70.36 | 116.46 | 1.65 | 23.55 | 31.01 | 0.91 | 17.55 | 17.44 | 0.00 | 634.36 | 636.69 | 0.03 |
| Yemen | 35.24 | 32.31 | -0.25 | 9.35 | 7.65 | -0.64 | 7.68 | 5.55 | -1.02 | 273.43 | 197.94 | -1.01 |
| Zambia | 47.51 | 76.52 | 1.59 | 17.50 | 27.20 | 1.43 | 14.76 | 21.34 | 1.20 | 530.18 | 774.77 | 1.23 |
| Zimbabwe | 38.58 | 49.68 | 0.90 | 11.54 | 15.95 | 1.08 | 8.91 | 12.51 | 1.14 | 315.83 | 457.54 | 1.24 |
